# Design, Synthesis and Evaluation of *N*-pyrazinylbenzamides as Potential Antimycobacterial Agents

**DOI:** 10.3390/molecules23092390

**Published:** 2018-09-18

**Authors:** Jan Zitko, Alžběta Mindlová, Ondřej Valášek, Ondřej Jand’ourek, Pavla Paterová, Jiří Janoušek, Klára Konečná, Martin Doležal

**Affiliations:** 1 Faculty of Pharmacy in Hradec Králové, Charles University, Heyrovského 1203, Hradec Králové 500 05, Czech Republic; alzbeta.mindlova@gmail.com (A.M.); valaseko@faf.cuni.cz (O.V.); jando6aa@faf.cuni.cz (O.J.); janousj2@faf.cuni.cz (J.J.); konecna@faf.cuni.cz (K.K.); dolezalm@faf.cuni.cz (M.D.); 2 Department of Clinical Microbiology, University Hospital, Sokolská 581, Hradec Králové 500 05, Czech Republic; pavla.paterova@fnhk.cz

**Keywords:** antibacterial, antifungal, antimycobacterial, cytotoxicity, linker, pyrazinamide, retro-amide, tuberculosis

## Abstract

Three series of *N*-(pyrazin-2-yl)benzamides were designed as retro-amide analogues of previously published *N*-phenylpyrazine-2-carboxamides with in vitro antimycobacterial activity. The synthesized retro-amides were evaluated for in vitro growth inhibiting activity against *Mycobacterium tuberculosis* H37Rv (*Mtb*), three non-tuberculous mycobacterial strains (*M. avium*, *M. kansasii*, *M. smegmatis*) and selected bacterial and fungal strains of clinical importance. Regarding activity against *Mtb*, most *N*-pyrazinylbenzamides (retro-amides) possessed lower or no activity compared to the corresponding *N*-phenylpyrazine-2-carboxamides with the same substitution pattern. However, the active retro-amides tended to have lower HepG2 cytotoxicity and better selectivity. Derivatives with 5-chloro substitution on the pyrazine ring were generally more active compared to their 6-cloro positional isomers or non-chlorinated analogues. The best antimycobacterial activity against *Mtb* was found in *N*-(5-chloropyrazin-2-yl)benzamides with short alkyl (**2h**: R^2^ = Me; **2i**: R^2^ = Et) in position 4 of the benzene ring (MIC = 6.25 and 3.13 µg/mL, respectively, with SI > 10). *N*-(5-Chloropyrazin-2-yl)benzamides with hydroxy substitution (**2b**: R^2^ = 2-OH; **2d**: R^2^ = 4-OH) on the benzene ring or their acetylated synthetic precursors possessed the broadest spectrum of activity, being active in all three groups of mycobacterial, bacterial and fungal strains. The substantial differences in in silico calculated properties (hydrogen-bond pattern analysis, molecular electrostatic potential, HOMO and LUMO) can justify the differences in biological activities between *N*-pyrazinylbenzamides and *N*-phenylpyrazine-2-carboxamides.

## 1. Introduction

According to the latest Global Tuberculosis Report published by the WHO [1], an estimated 10.4 million people worldwide developed active tuberculosis (TB) in 2016 and TB caused 1.7 million deaths, including 0.4 million deaths among HIV-positive people. This mortality rate ranks TB the leading cause of death from infectious diseases and in the top 10 causes of death generally, following cardiovascular and respiratory diseases [1]. A recent study estimated that 23% of global population (1.7 billion people) is latently infected with TB [2]. Multidrug-resistance is a serious epidemiologic issue connected with TB. As estimated for 2016, there were 490,000 new cases of multidrug-resistant TB (MDR-TB; resistant to rifampicin and isoniazid) and additionally 110,000 cases of rifampicin-resistant TB (RR-TB) [1]. Even the basic regimen for non-resistant TB consists of at least four first-line antitubercular drugs (rifampicin, isoniazid, ethambutol and pyrazinamide) administered for six months and it is challenging for patients’ compliance. The compliance problem is further deepened in MDR-TB, as the simplest MDR-TB regimen recommended by the WHO takes 6–9 months and requires parenteral application [3]. There is therefore an urgent clinical requirement for the development of new anti-TB drugs, leading to more effective and shorter therapeutic regimens.

Pyrazinamide (PZA; pyrazine-2-carboxamide) as a first-line antitubercular agent is an important component of the initial phase of all basic TB treatment regimen [1]. PZA, its metabolite pyrazine-2-carboxylic acid (POA), or their simple structure derivatives were shown to act as inhibitors of mycobacterial fatty acid synthase I (FAS I) [4,5,6,7,8], aspartate decarboxylase (PanD) [9,10,11], and quinolinic acid phosphoribosyltransferase (QAPRTase) [12]. Based on the results of protein binding assays and X-ray crystallography, POA was suggested to act as an inhibitor of trans-translation, the process of rescuing ribosomes stalled during translation [13,14]. However, this mechanism of action (MoA) was recently disputed [15]. The perception of PZA and its metabolite POA has changed from a non-specific cytosol acidifier to a multi-target inhibitor of specific enzymes and processes of mycobacteria [16].

**Figure 1:**
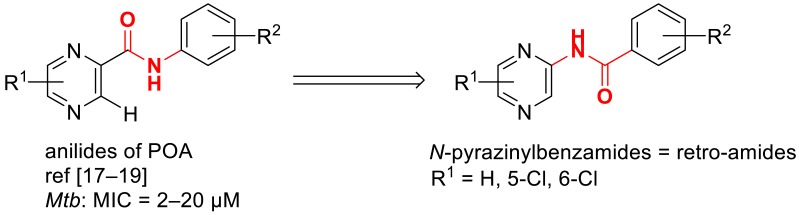
Design of title compounds inspired by previously published *N*-phenylpyrazine-2-carboxamides with antimycobacterial activity.

Previously published *N*-phenylpyrazine-2-carboxamides (in other words anilides of POA) with different simple substituents on the pyrazine ring (R^1^ is H, methyl, *tert*-butyl, chloro or combination thereof) and simple substituents on the benzene ring (R^2^ is short alkyl, hydroxy, halogen, nitro or combination thereof, Figure 1) possessed in vitro growth inhibiting activity against *Mycobacterium tuberculosis* H37Rv (*Mtb*) and other non-tuberculous mycobacterial strains. Best compounds possessed activity with MIC at micromolar level (2–20 µM). Structure-activity relationships (SAR) in this class were reviewed elsewhere [17,18,19]. Direct inspiration for compounds presented in this paper was our previous series of 5-chloro-*N*-phenylpyrazine-2-carboxamides [18], which were generally more active (best compounds with MIC at low micromolar level) than previously studied 6-Cl isomers. The benzene ring in the series tolerated different substituents, both electron-donating and electron-withdrawing, and hydrophilic and lipophilic. The drawback was significant in vitro cytotoxicity (HepG2) with IC_50_ for most compounds at units or tens of µM, leading to insufficient selectivity in most of the compounds. As a continued effort to extend the knowledge of SAR of *N*-phenylpyrazine-2-carboxamides as antimycobacterial agents, we designed a series of *N*-(pyrazin-2-yl)benzamides, which can be regarded retro-amide analogues (Figure 1).

Exchanging the amide moiety for a retro-amide is a strategy successfully used in medicinal chemistry and drug design. The obligatory textbook example is the group of local anesthetics. Cinchocaine is a substituted amide of a heteroaromatic acid, therefore having the -CONH- connecting bridge between the aromatic core and the basic amino moiety in the side chain. However, significantly more populated group of local anesthetics are anilides having the retro-amide -NHCO- linker (e.g., lidocaine, trimecaine, bupivacaine). In the field of antimicrobial research, the retro-amide modification led recently to derivatives with increased activity against *Candida albicans* [20]. The retro-amides of classical β-lactam antibiotics and their analogues were studied as substrates and/or inhibitors of beta-lactamases [21,22,23,24].

Thus, the main aim of our study was to evaluate the effect of amide (-CONH-) vs retro-amide (-NHCO-) linker between the pyrazine and benzene core on in vitro antimycobacterial activity. Along with the linker exchange, we kept the variability both in the pyrazine core (R^1^ is H, 5-Cl, or 6-Cl) and in the benzene core (R^2^). Synthetic work was performed by two undergraduate students (co-authors of this paper, A.M. and O.V.) as a part of their diploma theses, therefore we kept the chemistry complexity at a suitable level.

## 2. Results and Discussion

### 2.1. Chemistry

**Scheme 1:**
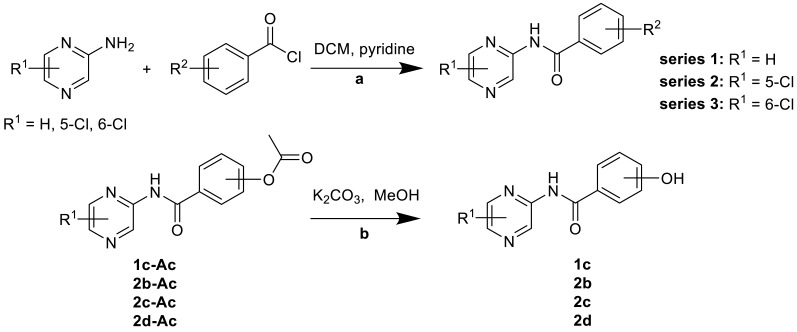
Synthesis of final compounds: (**a**) Benzoylation of aminopyrazine; (**b**) Hydrolysis of acetylated derivatives.

Final compounds were prepared by simple benzoylation (Scheme 1, **a**) of aminopyrazine (series **1**), 5-chloropyrazin-2-amine (series **2**), or 6-chloropyrazin-2-amine (series **3**). The source of the substituted benzoyls were commercially available benzoyl chlorides. In the case of hydroxy substituted derivatives, we used acetoxybenzoyl chlorides and consequently cleaved off the protecting acetyl in the product by mild alkaline hydrolysis of the ester bond (Scheme 1, **b**).

**Figure 2:**
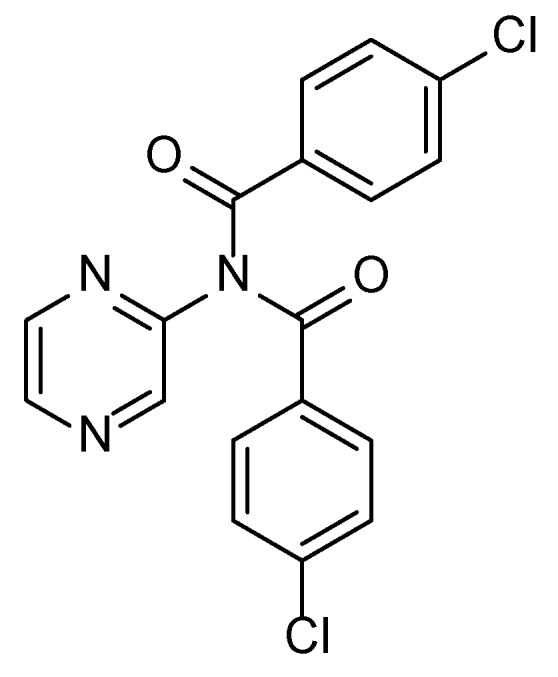
Structure of *N*,*N*-diacylated product **1l-SP**.

In the benzoylation step, we were concerned about possible diacylation, which would produce undesired side products by substitution of both hydrogens of the amino moiety of aminopyrazine. Such *N*,*N*-diacylation was described for aminopyridine, 2-aminopyrimidine, aminopyrazine and similar heterocyclic amines in a recent study [25]. In that study, the tendency for diacylation increased with the strength of used base, more specifically, employment of pyridine yielded dominantly monoacylated products, whereas triethylamine (Et_3_N) yielded dominantly diacylated products or mixtures [25]. Indeed, this was confirmed by our experiments, when method A described in Section 3.2.1 with 3 molar equivalents (equiv) of Et_3_N instead of pyridine led to diacylated product **1l**-SP (Figure 2). Using Et_3_N as a base, only diacylated products were obtained. Therefore, in all further reactions we used dry pyridine (3 or 4 equiv) as a base. To further decrease the probability of formation of diacylated products, the mixture of the benzoyl chloride with pyridine in appropriate solvent was pre-cooled in the freezer and subsequently cooled in an ice bath during addition of the aminopyrazine component to prevent spontaneous heating. We expected that increased reaction temperature could have preferred diacylation.

Final compounds were isolated as white or very pale yellow colored solids. The isolated yields of chromatographically pure product after all purification steps ranged from 8–67% for Method A and 43–85% for Method B. Method B is a modification of Method A and was performed under nitrogen atmosphere and with extended work-up. Method A performed without the inert atmosphere gave lower, but still acceptable yields.

The deprotection of acetoxy derivatives was achieved by mild base catalyzed hydrolysis of the acetate bond by dissolving in EtOH and heating with excess of K_2_CO_3_. However, in some cases, we observed spontaneous acetate hydrolysis during workup and/or purification. For example, in the attempt to prepare **1b-Ac**, the compound hydrolyzed during flash chromatography (this could have been catalyzed by the acidic properties of silica) or during recrystallization from hot aqueous ethanol.

All final products were fully characterized by melting point, ^1^H- and ^13^C-NMR spectra, IR spectra and elementary analysis. The acquired data were fully consistent with proposed structures. The summary or prepared compounds and their codes are presented in Table 1.

### 2.2. Antimycobacterial Activity

Title compounds were screened for in vitro growth inhibiting activity against *Mycobacterium tuberculosis* H37Rv (*Mtb*), two non-tubercular slow growing mycobacterial strains (*M. kansasii* and *M. avium*) and a strain of fast-growing *M. smegmatis*. The assays were Microplate Alamar Blue Assays (MABA) [26] with modifications. See Table 2 for active compounds and the Supplementary Material for full results and detailed experimental procedures.

Regarding the antimycobacterial activity against *Mtb*, series **2** derived from 5-Cl-pyrazin-2-amine was the most successful, containing five active compounds with MIC ranging 3.13–25 µg/mL. The activity in series **1** derived from non-chlorinated aminopyrazine as well as in series **3** derived from 6-Cl-pyrazin-2-amine was lower (MIC = 25–100 µg/mL) and less abundant, yielding four moderately or low active compounds in series **1** and two moderately or low active compounds in series **3** (see graphical representation in Table 1). The superiority of 5-chloropyrazine substituted derivatives is in concordance with relationships observed in previous series, where 5-chloro-*N*-phenylpyrazine-2-carboxamides [18] were more active than their 6-Cl positional isomers [17] or non-chlorinated derivatives [27].

Discussing the substituent R^2^ on the benzene ring, 2-OH derivative (**2b**) and its acetylated precursor (**2b-Ac**) showed MIC of 12.5 µg/mL against *Mtb*, and importantly showed similar activity on the non-tubercular mycobacterial strains. As it will be mentioned in Section 2.4, the activity spectrum of these two compounds expands also to tested fungal strains. Another successful substituent R^2^ in series **2** was short alkyl in position 4 (**2h**: R^2^ = 4-CH_3_; **2i**: R^2^ = 4-Et) with MIC = 3.13–6.25 µg/mL against *Mtb.* Substitution R^2^ = 3-CF_3_ yielded active compounds in both chlorinated series (compounds **2n** and **3n**). In the case of compound **3n**, the activity spectrum was broad and covered all tested mycobacterial strains. The most active derivative in series **1** was **1g** with R^2^ = 4-OCH_3_. Generally, the most active derivatives in all series **1**, **2** and **3** follow the substitution pattern that was proposed for *N*-phenylpyrazine-2-carboxamides [19], that is, the benzene ring substituted with electron-withdrawing substituent in position 3 and/or electron-donating substituent in position 4.

### 2.3. Antibacterial Activity

As a complementary test, final compounds were screened for in vitro antibacterial activity against eight G+ and G− bacterial strains of clinical importance. The method was a microplate dilution assay with MICs determined by naked eye. For experimental details, list of tested strains and full results see Supplementary Material. Table 3 lists compounds with antibacterial MIC ≤ 125 µM (and some of their inactive positional isomers for comparison). It is evident that title compounds exerted mainly anti-staphylococcal activity and that (with the exception of **1n**) they belonged to series **2** derived from 5-Cl-pyrazin-2-amine. Significant activity was exerted by hydroxy derivatives **2b**, **2d** and their acetylated precursors **2b-Ac**, **2d-Ac**. We assume that acetylated derivatives might act as transport form and undergo hydrolysis by various esterases either in plasma or after penetration to a bacterial cell. Interestingly, the hydroxy or acetoxy substituent could be in position 2 or 4 of the benzene ring, but not in position 3—compounds **2c** and **2c-Ac** were inactive. 2-Substituted derivatives **2b** and **2b-Ac** inhibited all three tested *Staphylococcus* strains (including MRSA), whereas 4-substituted derivatives **2d** and **2d-Ac** were active only against the reference strain of *S. aureus*. Positional isomerism on benzene ring effected the activity also in other examples—the 2-Cl derivative **2j** and 3-OCH_3_ derivative **2f** showed moderate activity against *S. aureus*, but their positional isomers **2k**, **2l** and **2e**, **2g** were inactive (MIC > 125 µM). Most compounds of series **3** (derived from 6-Cl-pyrazin-2-amine) could have not been tested due to excessive precipitation in the testing medium.

### 2.4. Antifungal Activity

As a complementary test, final compounds were tested for in vitro antifungal activity against eight species of clinical importance. The testing was based on microdilution method; results are expressed as MIC (µM) read by naked eye. For experimental details, list of tested strains and full results see Supplementary Material. The following paragraph concerns the MIC values read after 24 h of incubation (with the exception of *Trichophyton interdigitale*, where the first reading was after 72 h‎).

In series **2** (R^1^ = 5-Cl), compound **2b** (R^2^ = 2-OH) exerted moderate and strain non-specific antifungal activity, inhibiting all tested strains with MIC = 62.5 or 125 µM. Compound **2b-Ac**, the acetylated precursor of **2b**, showed similarly broad spectrum of activity of moderate-low level (MIC = 125–250 µM). It is probable that **2b** is generated by hydrolysis of acetate **2b-Ac** spontaneously (cultivation at 35 °C in water-based medium) and/or by the pathogen metabolism. Interestingly, the activity seems to be strongly dependent on the position of the hydroxyl substituent, as 3-OH (**2c**) and 4-OH (**2d**) positional isomers were inactive (MIC ≥ 500 µM). Compound **2f** (R^2^ = 3-OCH_3_) showed moderate activity against *Candida albicans* (MIC = 125 µM) but no activity against any other tested fungal species. The rest of the tested compounds from series **2** were inactive up to the highest tested concentration, which (dependent on the solubility) were 125 µM or 500 µM. The antifungal activity determined for the title compounds must be considered insignificant in comparison with standard nystatin, which expressed MIC ranging from 0.98 to 15.62 µM for all tested strains (see Supplementary Material for MIC values of additional standards). No antifungal activity was observed in series **1** and **3**. Most of the compounds of series **3** (derived from 6-Cl-pyrazin-2-amine) could not be tested due to excessive precipitation in the testing medium. See Supplementary Material for full results in tabular form.

### 2.5. In Vitro Cytotoxicity

Selected active compounds were tested for their in vitro cytotoxic effect in human hepatocellular carcinoma cell line (HepG2), using a commercial colorimetric assay (CellTiter 96^®^ AQueous One Solution Cell Proliferation Assay, Promega, Madison, WI, USA). The cells were incubated with different concentrations of tested compounds and after 24 h, the viability was compared to untreated cells. All experiments were performed in triplicates. Viability curves were constructed, and the cytotoxicity was expressed as IC_50_, that is, the concentration required to decrease the viability of cell population to 50% compared to the control of 100% cell viability. See Table 4 for results. The limited solubility of compounds **2d-Ac**, **2h**, **2i** and **3n** in the culture medium did not allow for the measurement at sufficiently high concentrations, therefore the viability curve could not be constructed. In other words, the IC_50_ of these compounds is significantly higher than the highest measured concentration (250 μM or 500 μM). To assess the selectivity to (myco)bacterial cells over human cells, we calculated the corresponding selectivity indexes (Table 4). Compounds with SI > 10 are considered to have potential for further development.

As a confirmatory test, **2b** and **2i** were further tested for prolonged cytotoxic effects on HepG2 cells using commercial fluorescence kit CellTox™ Green Cytotoxicity Assay (Promega). The IC_50_ values were determined after 24 h and 48 h of incubation with tested compounds—see Table 5 for results. The results confirmed low cytotoxicity with IC_50_ values at hundreds of µM. Importantly, the cytotoxicity of **2i** did not increase with time. Experimental details for both cytotoxicity tests can be found in the Supplementary Material.

### 2.6. Comparison of the Antimycobacterial Activity and HepG2 Cytotoxicity of N-Pyrazinylbenzamides (Retro-Amides) with the Corresponding N-Phenylpyrazine-2-carboxamides (Amides)

The central question of our study was how the inversion of the amide linker in the general structure of previously published *N*-phenylpyrazine-2-carboxamides will influence their antimycobacterial activity. To assess this issue, we compared the antimycobacterial activity of title *N*-pyrazinylbenzamides (retro-amides, -NHCO-, left side of Table 6) of this study to the activity of previously published counterparts (-CONH-, right side of Table 6) with the same substitution both on the pyrazine (R^1^) and benzene (R^2^) ring. As the activity on other strains was rather sporadic, we focused on the activity against *Mtb*. 

As indicated in Table 6, for most of the pairs, the activity of *N*-pyrazinylbenzamides (retro-amides) was substantially lower or none in comparison with original *N*-phenylpyrazine-2-carboxamides. A significant exception was **2i** (R^1^ = 5-Cl, R^2^ = 4-Et), the most active derivative of this study, whose activity (MIC = 3.13 µg/mL) against *Mtb* was comparable with its *N*-phenylpyrazine-2-carboxamide counterpart (MIC = 0.78 or 1.56 µg/mL). The evaluation of linker inversion effect in series **1** (R^1^ = H) and **3** (R^1^ = 6-Cl) is complicated by the fact that for many of the corresponding amides, the MIC values were not determined and the only value we have for comparison is the percentage of growth inhibition at fixed concentration 6.25 µg/mL. (These results were produced in the Tuberculosis Antimicrobial Acquisition and Coordinating Facility (TAACF) screening campaign [28]). In case that the retro-amide is completely inactive (MIC > 100 µg/mL), the direct comparison is possible. In other cases, where there is some activity for the retro-amide and some percentage of inhibition for the amide, the comparison should be taken as a rough guess, as indicated with the question mark in Table 6.

Generally, it can be concluded that inversion of the amide linker in *N*-phenylpyrazine-2-carboxamides leads to decrease or loss of antimycobacterial activity in most cases.

*N.B.*: The methodologies used for MIC determination of historically published compounds (amides) were similar, but not identical, to conditions used in the up-to-date testing used in this paper. The differences are stated as footnotes to Table 6 and details can be found in the original literature as referenced. It is well known that the growth of mycobacteria is highly influenced by the conditions of cultivation, for example pH [29,30]. Therefore, the comparisons presented in the preceding paragraphs and in Table 6 are to be taken as an assessment of general trends and not as comparison of precise MIC values. 

Focusing on the in vitro HepG2 cytotoxicity, the template ‘amides’ exerted significant toxicity with IC_50_ for most compounds at units or tens of µM, leading to insufficient selectivity in most of the compounds [18]. The IC_50_ values for here presented retro-amides were in hundreds of µM. Direct comparison of identically substituted pairs of amide vs retro-amide analogues are presented in Table 7. Although based on limited data, it can be implicated that the exchange of amide for retro-amide linker decreases the HepG2 cytotoxicity 10 times.

### 2.7. In Silico Prediction of Molecular Structure and Properties

As we showed in the previous section, it is obvious that exchange of an amide linker for a retro-amide linker has profound effects on the biological activity of the pyrazine-linker-benzene-like compounds. In an attempt to rationalize the differences, we selected a pair constituted by the inactive retro-amide **2a** and its active amide counterpart for an in silico study of their molecular structure and properties, which could help explain possible differences in binding to a hypothetical molecular receptor. Molecular mechanics (AMBER10:EHT forcefield) and quantum mechanics (B3LYP/cc-pVTZ level of theory) were used to predict the energetically minimized conformations. Molecular electrostatic potential (MEP) and frontier orbitals (HOMO, LUMO) were calculated at the B3LYP/cc-pVTZ level of theory.

#### 2.7.1. Geometry Optimization

**Figure 3:**
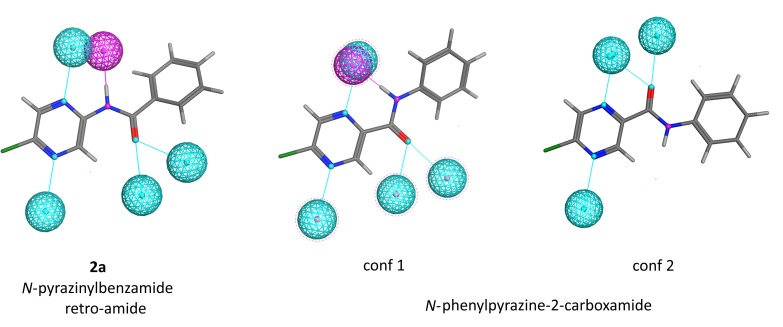
Analysis of selected pharmacophore features of the minimized conformations. Turquoise dot—HBA, violet dot—HBD. Spheres represent the respective projections.

**Figure 4:**
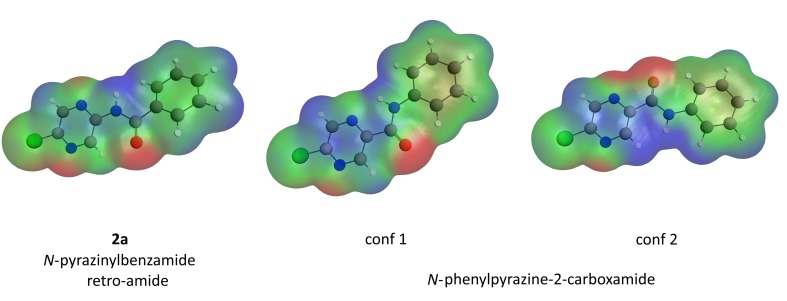
Total electron density isosurface colored by molecular electrostatic potential (MEP).

In the structure optimization efforts, we realized that the amide counterpart has two sterically significantly different conformers of similar energy (see Figure 3 and Figure 4)—conf 1 with E_h_ = −1123.499 hartree and conf 2 with E_h_ = −1123.483 hartree as predicted on B3LYP/cc-pVTZ level of theory. The two conformers both have the plane of the amide bond in the plane of the pyrazine ring but differ by rotation of 180 degrees. The presence of two energetically close conformers of *N*-phenylpyrazine-2-carboxamides was predicted by QM calculations before [36]. With such close energy values, it is presumable that both of the presented conformers are biologically relevant.

#### 2.7.2. Analysis of Pharmacophore Features

We analyzed the hydrogen-bonding pattern that the compounds are able to produce in their minimized conformations. Pharmacophore features (HBD, HBA and their projections) are presented in Figure 3. Projections are depicted as spheres, which represent the space where the heavy atom of the bonding partner of the protein should be present. There were significant differences among the studied compounds/conformers. Both retro-amide **2a** and amide in conf 1 have three HBAs (which produce in total four HBA projections) and one HBD. In **2a**, the projection sphere of the HBA arising from the N1 of the pyrazine ring is close to the HBD projection coming from the -NH- moiety of the linker. It might be difficult for the protein to satisfy both of these demands due to steric hindrance. In conf 1, the situation is similar, but the projection spheres of the HBA and HBD are overlapping. This means that one functional group (let us say a hydroxyl) of a protein could satisfy both the HBA and HBD features of the ligand at the same time. Amide in conf 2 has only three HBAs and no HBD projection. Of course the potential HBD in the form of -NH- of the linker is still present, but its projection sphere is occupied by H-3 of the pyrazine core, so in this conformation the HBD cannot interact with protein. We can conclude that *N*-phenylpyrazine-2-carboxamide prototype (conf 1) should have the greatest potential for interaction with a theoretical protein receptor.

#### 2.7.3. Molecular Electrostatic Potential (MEP)

Total electron density isosurface corresponding to the van der Waals size of the molecule (isosurface contour 0.002 au) was calculated and mapped with molecular electrostatic potential (MEP, Figure 4). Red color represents the most negative potential (places with relative abundance of electrons), whereas the positive potential (relative lack of electrons) is represented by blue color. In between values are colored according to the RGB scale (green representing neutral values with MEP close to zero). From the MEP, it is evident that the negative charge covers the carbonyl group and the positive region is over the -NH- group of the linkers. High electronegativity of the carbonyl group makes it the most reactive part of the molecule in all studied compounds/conformers. Direct comparison of retro-amide **2a** with amide in conf 1 reveals the differences in the electron density in the pyrazine nucleus. In **2a**, both N-1 and N-4 of the pyrazine ring are associated with negative MEP potential and therefore are expected to be strong HBAs. The amide in conf 1, however, lacks significantly negative MEP at N-1. This can be explained by close vicinity of the carbonyl with electron-withdrawing properties. We can conclude that the ring N-1 in amide in conf 1 is expected to be weaker HBA compared to N-1 of the retro-amide. Amide in conf 2 is different to conf 1 and creates a large area of negative potential around carbonyl and N-1, and a large area of positive potential around -NH- of the linker and H-3 of the pyrazine.

#### 2.7.4. HOMO and LUMO Orbitals

**Figure 5:**
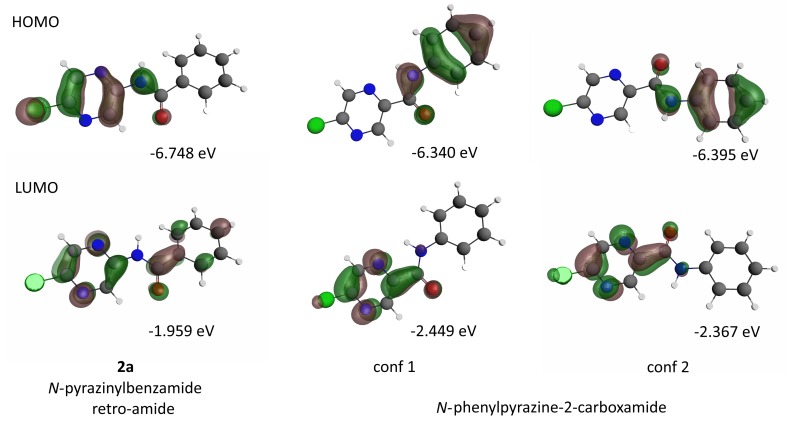
Predicted HOMO and LUMO.

The HOMO and LUMO orbitals were calculated at the B3LYP/cc-pVTZ level of theory (Figure 5). The spatial distributions of both HOMO and LUMO were very similar between conf 1 and conf 2 of the amide, so they will be discussed jointly. HOMO of **2a** (retro-amide) was localized on the pyrazine core and on the linker, whereas HOMO of the amide was on the phenyl and the linker. LUMO of **2a** was localized over the whole molecule, whereas in amide the LUMO was restrained to the pyrazine and the linker. The HOMO-LUMO gap was 4.789 eV for **2a**, 3.891 eV for amide in conf 1, and 4.027 eV for amide in conf 2. Without further detailed analysis, it can be stated that the exchange of amide linker for retro-amide linker has profound effects on the localization of the frontier orbitals (HOMO and LUMO) and is therefore probable to influence compounds reactivity and interactions to a hypothetic receptor.

## 3. Materials and Methods

### 3.1. General

All reagents and solvents (unless stated otherwise) were purchased from Sigma-Aldrich (Schnelldorf, Germany). Aminopyrazine (98%, Sigma-Aldrich), and 6-chloropyrazin-2-amine (97%, Synthonix, Wake Forest, NC, USA) were used without any purification. 5-Chloropyrazin-2-amine (>95%, Synthonix) as purchased was contaminated by a significant amount of another compound, probably a positional isomer as indicated by NMR. Therefore, a routine flash chromatography (silica, EtOAc in hexane gradient elution) purification was applied before usage. Substituted benzoyl chlorides were purchased from Sigma-Aldrich.

The reaction process and the purity of final compounds were checked using Silica 60 F_254_ TLC plates (Merck, Darmstadt, Germany). Flash chromatography of the final compounds was performed on a CombiFlash Rf 200 automated chromatograph (Teledyne Isco, Lincoln, NE, USA) using columns filled with Kieselgel 60, 0.040–0.063 mm (Merck) and a detection wavelength of 280 nm. NMR spectra were recorded on Varian VNMR S500 or at Varian Mercury VX-BB 300 spectrometers (Varian, Palo Alto, CA, USA). The spectra were recorded in DMSO-*d_6_* or CDCl_3_ at ambient temperature. The chemical shifts as δ values in ppm are indirectly referenced to tetramethylsilane (TMS) via the solvent signal. IR spectra were recorded on a Nicolet Impact 400 spectrophotometer (Nicolet, Madison, WI, USA) using ATR-Ge method. Elemental analysis was performed on a Vario MICRO cube Element Analyzer (Elementar Analysensysteme, Hanau, Germany). All values regarding elemental analyses are given as percentages. Melting points were determined in open capillary on Stuart SMP30 melting point apparatus (Bibby Scientific Limited, Staffordshire, UK) and are uncorrected. Yields are given as percentages and refer to the amount of chromatographically pure product after all purification steps.

### 3.2. Chemistry

#### 3.2.1. Method A (Used in the Synthesis of Final Products from Series **1** and **3**)

A mixture of dry dichloromethane (DCM, 2 mL) and dry pyridine (475 mg, 6 mmol, 3 molar equiv) was put into 25 mL round-bottom flask, closed with a stopper and cooled in a freezer for approximately 15 min. A selected benzoyl chloride (2.4 mmol, 1.2 equiv) was diluted with dry DCM (5 mL) and added dropwise to the cooled (ice bath) pyridine/DCM mixture under stirring, and the mixture was stirred for additional 5 min in the closed flask. 2-Aminopyrazine (190 mg, 2 mmol, 1 equiv) or 6-chloropyrazin-2-amine (259 mg, 2 mmol, 1 equiv) was dissolved in DCM (2 mL) and added dropwise to the cooled reaction mixture over 10 min upon stirring. After additional 15 min, the reaction was removed from the ice bath and stirred at laboratory temperature. The progress of reaction was monitored by TLC (silica plates, 33% EtOAc in hexane). After 2 h, no significant further increase in the spot of the product was observed, so the reaction was ended and worked-up.

The reaction mixture was adsorbed on silica (4 g) by evaporating the solvents under reduced pressure. The mixture on silica was used for solid loading the flash chromatography pre-column. The separation used the following conditions: manually filled silica column (30 g), continuous gradient elution 0–50% EtOAc in hexane, flow rate 35 mL/min, detection wavelength 280 nm, monitoring wavelength 260 nm. Fractions containing pure product were combined and solvents were evaporated under reduced pressure to yield solid product. If needed, the products were recrystallized from hot EtOH, the crystallization was induced by cooling and addition of water. The products were isolated as white solids. In several cases, the final products were still contaminated with non-specified impurity of brown color. This impurity was easily removed by dispersing the product in small amount of hexane and immersion of a vertical piece of filtration paper into this dispersion. The impurity was soluble in hexane and rose by capillary action to the filtration paper.

#### 3.2.2. Method B (Used in the Synthesis of Final Products from Series **2**) 

Method B is a modification of Method A and was performed under nitrogen atmosphere and with extended work-up. Substituted benzoyl chloride (1.5 mmol, 1.2 equiv) was placed into the flask under nitrogen, diluted with dry DCM (5 mL) and dry pyridine (400 mg, 5 mmol, 4 equiv) was added. The mixture was mixed for 5 min under nitrogen. Then, 5-chloropyrazin-2-amine (162 mg, 1.25 mmol, 1 equiv) dissolved in DCM (10 mL) was added dropwise over 10 min under nitrogen flow. The flask was closed by septum and stirred for additional 6 h. After reaction, the mixture was diluted with DCM to the final volume of 40 mL and washed with water (1 × 30 mL), 5% (*m*/*m*) aqueous NaHCO_3_ solution (1 × 30 mL), and brine (1 × 30 mL). The organic layer was dried over anhydrous Na_2_SO_4_ and adsorbed on silica (4 g) by evaporating the solvents under reduced pressure. Automated flash chromatography was run using same conditions as described in Method A. If needed, the products were recrystallized from hot EtOH (crystallization initiated by cooling and dropwise addition of cold water).

#### 3.2.3. Hydrolysis of Acetates

The acetylated product (compounds **1c-Ac**, **2b-Ac**, **2c-Ac**, or **2d-Ac**) was placed in a beaker and dissolved in hot aqueous MeOH. Excess (5 equiv) of potassium carbonate (K_2_CO_3_) was added and the reaction mixture was stirred at 50 °C for 2 h under TLC control. Reaction mixture was adsorbed on silica and purified using flash chromatography (silica, gradient elution EtOAc in hexane 10–60%).

### 3.3. Analytical Data of Prepared Compounds

Analytical data of prepared compounds, including the references to previously published compounds, are located in the Supplementary Material.

### 3.4. Biological Methods

For tested strains and detailed description of the biological methods, refer to the Supplementary Material.

### 3.5. In Silico Calculations

Energetically minimized 3D structures of **2a** and its amide counterpart were predicted by MOE v2018.1001 (Molecular Operating Environment, Chemical Computing Group Inc., Montreal, QC, Canada). All calculations were performed under AMBER10:EHT forcefield (implicit R-field solvent model). The structures were drawn manually in MOE Builder and initially minimized (the built-in minimization method combines both steepest gradient and conjugate gradient methods) to 0.1 kcal·mol^−1^·A^−2^. These minimized conformations were subjected to conformational search using low mode molecular dynamics with default settings to determine the global minimum for each compound. The resulting conformer with lowest energy for each compound was considered a representative structure. The obtained coordinates were used to prepare the input for DFT calculations, which were performed in GAMESS (version 2016-pgi-linux-mkl, Mark Gordon’s Quantum Theory Group, Ames Laboratory/Iowa State University, Ames, IA, USA). In GAMESS, the geometry of the structures was first optimized (RUNTYP = OPTIMIZE, SCF gradient convergence criteria 0.001 Hartree/Bohr) and subsequently the single point energy of the minimized structures was calculated (RUNTYP = ENERGY). All QM calculations were performed at B3LYP/cc-pVTZ level of theory, which was previously used for *N*-phenylpyrazine-2-carboxamides and gave predictions consistent with experimentally determined properties [36]. Figure 3 was generated in MOE, using the Pharmacophore Editor and Unified annotation scheme of pharmacophore features. Figure 4 and Figure 5 were generated in wxMacMolPlt v7.7 (freeware by Brett Bode, NCSA, Urbana, IL, USA) [37].

## 4. Conclusions

In this study, we have designed and prepared 41 *N*-(pyrazin-2-yl)benzamides, which can be regarded as retro-amide analogues of previously studied antimycobacterial *N*-phenylpyrazine-2-carboxamides. To our knowledge, 35 of the reported compounds are new, not previously described in literature (as checked in CAS SciFinder on 27 July 2018). Compounds **1d-Ac**, **1g**, **1h**, **1j**, and **1n** were previously described in literature (see the Analytical Data section in Supplementary Material), but nothing had been published on their antimycobacterial or generally anti-infective activity. Compound **1b** was studied for its antimycobacterial properties in vitro [38] and the previously reported results are in concordance with our data.

Prepared retro-amides were tested for in vitro growth inhibiting activity on *Mtb* and three other mycobacterial strains. Our results clearly indicate that inversion to retro-amide linker usually leads to decrease or loss of activity, but the remaining active retro-amides are more selective and probably also less toxic to mammalian cells (HepG2) in comparison with the pattern amides (*N*-phenylpyrazine-2-carboxamides). The antimycobacterial activity appeared dominantly in series **2** derived from 5-chloropyrazin-2-amine (in concordance with previous observations that 5-Cl-*N*-phenylpyrazine-2-carboxamides were generally more active than their 6-Cl positional isomers or non-chlorinated analogues [18]). Among the prepared retro-amides of series **2**, significant antimycobacterial activity was found only for several compounds with specific substitution pattern on the benzene ring. This is in sharp contrast with the previously published 5-chloro-*N*-phenylpyrazine-2-carboxamides (amides), in which the benzene core tolerated many substituents, both hydrophilic and lipophilic and electron-donating and withdrawing [18].

Considering their MIC values, in vitro cytotoxicity (HepG2) and the resulting selectivity for mycobacterial cells over mammalian cells, the most promising *N*-pyrazinylbenzamides were **2h** and **2i,** that is, derivatives with short alkyl in position 4 of the benzene ring (see Table 4).

*N*-Pyrazinylbenzamides with hydroxy substitution on the benzene ring (such as **2b**) or their acetylated precursors (such as **2d-Ac**) exerted significant activity against *S. aureus* and sufficient selectivity over mammalian cells (Table 4). Compound **2b** (R^2^ = 2-OH) and its acetylated precursor **2b-Ac** (R^2^ = 2-OAc) had the broadest spectrum of activity, covering *Mtb*, non-tubercular mycobacterial strains, *S. aureus* and all the tested fungal strains. To explain the significant and broad-spectrum antimicrobial properties of **2b** and **2b-Ac**, one should consider that structurally they are salicylanilides, which are a class of compounds with well-known and repeatedly reported antimicrobial activity [39,40].

In silico calculations were performed to predict the hydrogen-bonding pattern, molecular electrostatic potential (MEP), and HOMO and LUMO orbitals for **2a** (as a prototype of the retro-amide) and compared to its amide counterpart. The observed differences can explain different levels of biological activity.

It can be concluded that the inversion of the amide linker (-CONH-) in *N*-phenylpyrazine-2-carboxamides to the retro-amide linker (-NHCO-) usually leads to a loss or decrease of in vitro antimycobacterial activity, but the resulting active retro-amides are more selective and have tighter SAR relationships regarding the benzene ring substitution. Some of the presented compounds with feasible activity and selectivity (**2h**, **2i** for *Mtb*; **2b**, **2d-Ac** for *S. aureus*) may become useful starting points for further development of antimicrobial compounds.

## Figures and Tables

**Table 1:** Summary of prepared derivatives—code assignment and activity against *Mtb.* Green—MIC = 3.13–6.25 µg/mL; Yellow—MIC = 12.5–25 µg/mL; Red—MIC = 50–100 µg/mL; White—MIC > 100 µg/mL (not active); n.a. = compound not available (not prepared).

	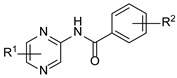		
	**R^1^**		
**R^2^**	H	5-Cl	6-Cl
H	**1a**	**2a**	**3a**
2-OH	**1b**	**2b**	n.a.
3-OH	**1c**	**2c**	n.a.
4-OH	n.a.	**2d**	n.a.
2-OCH_3_	**1e**	**2e**	**3e**
3-OCH_3_	**1f**	**2f**	**3f**
4-OCH_3_	**1g**	**2g**	**3g**
4-CH_3_	**1h**	**2h**	**3h**
4-Et	**1i**	**2i**	**3i**
2-Cl	**1j**	**2j**	**3j**
3-Cl	**1k**	**2k**	**3k**
4-Cl	**1l**	**2l**	**3l**
4-Br	n.a.	**2m**	n.a.
3-CF_3_	**1n**	**2n**	**3n**
2-OAc	n.a.	**2b-Ac**	n.a.
3-OAc	**1c-Ac**	**2c-Ac**	n.a.
4-OAc	**1d-Ac**	**2d-Ac**	n.a.

**Table 2:** Antimycobacterial activity of prepared compounds expressed as MIC (µg/mL) ^a^.

Cpd	R^1^	R^2^	MW	*Mtb* H37Rv ATCC 27294 MIC (µg/mL)	*M. kans.* ATCC 12478 MIC (µg/mL)	*M. avium* ATCC 15769 MIC (µg/mL)	*M. smeg.* ATCC 607 MIC (µg/mL)
**1a**	H	H	199.21	100	>100	>100	≥500
**1e**	H	2-OCH_3_	229.24	>100	100	>100	125
**1g**	H	4-OCH_3_	229.24	25	>100	>100	≥500
**1j**	H	2-Cl	233.66	>100	>100	>100	250
**1k**	H	3-Cl	233.66	50	50	>100	≥500
**1l**	H	4-Cl	233.66	50	50	>100	≥500
**1l-SP ^b^**	H	4-Cl ^b^	372.21	50	>100	>100	≥500
**2b-Ac**	5-Cl	2-OAc	291.69	12.5	12.5	50	31.25
**2b**	5-Cl	2-OH	249.65	12.5	50	50	15.625
**2c-Ac**	5-Cl	3-OAc	291.69	>100	>100	>100	250
**2d-Ac**	5-Cl	4-OAc	291.69	>100	>100	>100	250
**2h**	5-Cl	4-CH_3_	247.68	6.25	>100	>100	≥500
**2i**	5-Cl	4-Et	261.71	3.13	>100	>100	≥500
**2n**	5-Cl	3-CF_3_	301.65	25	>100	>100	≥500
**3f**	6-Cl	3-OCH_3_	263.68	50 ^c^	100 ^c^	100 ^c^	≥500 ^c^
**3n**	6-Cl	3-CF_3_	301.65	25	25	50	125
**INH**	-	-	137.14	0.1–0.39	6.25–12.5	6.25–12.5	15.625
**RFM**	-	-	822.95	-	-	-	1.56
**CPX**	-	-	331.35	-	-	-	0.195

^a^ Compounds not listed in the table were inactive against all tested strains. Complete version of the table is located in the Supplementary Material. ^b^ Diacylated side-product. ^c^ Precipitate formed in the testing medium. INH—isoniazid; RFM—rifampicin; CPX—ciprofloxacin.

**Table 3:** Antibacterial activity of selected compounds expressed as MIC (µM) in comparison with standard neomycin.

Strain	Time	Compound (Code, R^2^)									
		1n	2b-Ac	2b	2c-Ac	2c	2d-Ac	2d	2f	2j	Neom
		3-CF_3_	2-OAc	2-OH	3-OAc	3-OH	4-OAc	4-OH	3-OCH_3_	2-Cl	-
SA	24 h	>125	62.5	15.62	>125	500	0.98	31.25	31.25	62.50	3.90
	48 h	>125	125	15.62	>125	500	0.98	31.25	31.25	62.50	3.90
MRSA	24 h	>125	125	62.50	>125	>500	>125	>500	>500	>500	0.98
	48 h	>125	250	62.50	>125	>500	>125	>500	>500	>500	0.98
SE	24 h	62.50	125	62.50	>125	>500	>125	500	>500	>500	3.90
	48 h	62.50	250	62.50	>125	>500	>125	500	>500	>500	7.81

SA–*Staphylococcus aureus* CCM 4516/08; MRSA–*S. aureus* H 5996/08 methicillin resistant, clinical isolate; *S. epidermidis* H 6966/08, clinical isolate. Neom–neomycin. For more standard drugs, see Supplementary Material.

**Table 4:** In vitro HepG2 cytotoxicity of selected *N*-pyrazinylbenzamides expressed as IC_50_ (µM) and their selectivity indexes (SI) for *Mtb* and *S. aureus*.

Cpd	HepG2	*Mtb*		SA	
	IC_50_ (μM)	MIC (μM)	SI	MIC (μM)	SI
**2b-Ac**	95.37	42.85	2	65.20	1
**2b**	155.30	50.07	3	15.62	10
**2d-Ac**	>500 *	inactive	n.a.	0.98	>510
**2h**	>250 *	25.23	>10	>125	n.a.
**2i**	>250 *	11.96	>21	>125	n.a.
**3n**	>250 *	82.88	>3	n.t.	n.a.

* Measurement at higher concentrations was not possible due to the precipitation of the tested compound in testing medium. SI—calculated as IC_50_ /MIC in molar concentrations rounded to units. SA—*Staphylococcus aureus* CCM 4516/08, MIC after 24 h.

**Table 5:** Prolonged cytotoxicity in HepG2 cells.

Compound	IC_50_ (µM) after 24 h Exposure	IC_50_ (µM) after 48 h Exposure	Range of Tested Concentrations (µM)
**2b**	545.3	187.6	10–2000
**2i**	264.3	250.0	1–1000

**Table 6:** Comparison of antimycobacterial activity (*Mtb*) of prepared *N*-pyrazinylbenzamides (retro-amides) with corresponding *N*-phenylpyrazine-2-carboxamides (amides).

R^1^	R^2^	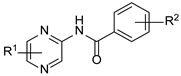 Retro-Amides		Relation	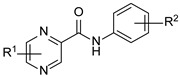 Amides	
		Code	MIC ^a^ (μg/mL)		MIC (μg/mL)	Inhibition (%) at 6.25 μg/mL (TAACF)
H	H	**1a**	100	=	>100 ^b^ [27]	0 [31]
H	2-OCH_3_	**1e**	>100	=	>100 ^b^ [27]	
H	3-OCH_3_	**1f**	>100	=	>100 ^b^ [27]	
H	4-OCH_3_	**1g**	25	<	>100 ^b^ [27]	
H	4-CH_3_	**1h**	>100	<	100 ^b^ [27]	86 [32]
H	2-Cl	**1j**	>100	<	50 ^c^ [19]	
H	3-Cl	**1k**	50	=?	>100 ^b^ [27]	14 [33]
H	4-Cl	**1l**	50	>=?	50 ^b^ [27]	4 [33]
H	3-CF_3_	**1n**	>100	<	6.25 [32], 50 ^b^ [27]	99 [32]
5-Cl	H	**2a**	>100	<	3.13 (1.56) ^c^ [18]	
5-Cl	2-OH	**2b**	12.5	<	3.13 (0.78) ^c^ [18]	
5-Cl	3-OH	**2c**	>100	<	6.25 ^c^ [18]	
5-Cl	4-OH	**2d**	>100	<	3.13 (12.5) ^c^ [18]	
5-Cl	4-Et	**2i**	3.13	=	1.56 (0.78) ^c^ [18]	
5-Cl	2-Cl	**2j**	>100	<	3.13 (0.78) ^c^	
5-Cl	3-Cl	**2k**	>100	<	6.25 (3.13) ^c^ [18]	
5-Cl	4-Br	**2m**	>100	<	3.13 ^c^ [18]	
5-Cl	3-CF_3_	**2n**	25	<	3.13 (6.25) ^c^ [18]	
6-Cl	H	**3a**	>100	<	25 ^d^ [34]	32 [31]
6-Cl	2-OCH_3_	**3e**	>100	<?		6 [35]
6-Cl	3-OCH_3_	**3f**	50	>?		2 [31]
6-Cl	4-CH_3_	**3h**	>100	<		71 [32]
6-Cl	2-Cl	**3j**	>100	<	100 ^d^ [34]	
6-Cl	3-Cl	**3k**	>100	<		14 [33]
6-Cl	4-Cl	**3l**	>100	<		65 [33]
6-Cl	3-CF_3_	**3n**	25	<		77 [32]

Conditions for determination of MIC: ^a^ Middlebrook 7H9 with OADC growth supplement, pH = 6.6, MABA; ^b^ Middlebrook 7H9, pH = 6.6, MABA; ^c^ Šula’s semisynthetic medium, pH = 5.6, growth indicated by turbidity; ^d^ Šula’s semisynthetic medium, pH = 5.6, MABA. TAACF—Tuberculosis Antimicrobial Acquisition and Coordinating Facility screening campaign. In Relation column—‘?’ indicates a rough guess.

**Table 7:** Comparison of in vitro HepG2 cytotoxicity of prepared *N*-pyrazinylbenzamides (retro-amides) with corresponding *N*-phenylpyrazine-2-carboxamides (amides).

Substituents		Retro-Amide -NHCO-		Amide -CONH-
R^1^	R^2^	Code	IC_50_ (μM)	IC_50_ (μM)
5-Cl	2-OH	**2b**	155.3	30.0 [18]
5-Cl	4-Et	**2i**	>250	7.2 [18]

## Supplementary Materials

Supplementary Material associated with this article can be found, in the online version, at doi: insert doi. Contents: Full procedures of the biological assays. Full results of biological assays. Analytical data or prepared compounds.
