# Supplementary material for: Design, Synthesis and Evaluation of N-pyrazinylbenzamides as Potential Antimycobacterial Agents
Source: Molecules. 2018 Sep 18;23(9):2390. doi: 10.3390/molecules23092390 (PMC6225228; doi:10.3390/molecules23092390)
Supplement: Supplementary file 1 [file molecules-23-02390-s001.pdf]

Article

Design, Synthesis and Evaluation of *N*-pyrazinylbenzamides as Potential Antimycobacterial Agents

Jan Zitko [1,*], Alžběta Mindlová [1], Ondřej Valášek [1], Ondřej Janďourek [1], Pavla Paterová [2], Jiří Janoušek [1], Klára Konečná [1], Martin Doležal [1]

[1] Faculty of Pharmacy in Hradec Králové, Charles University, Heyrovského 1203, Hradec Králové, 500 05, Czech Republic

[2] Department of Clinical Microbiology, University Hospital, Sokolská 581, Hradec Králové, 500 05, Czech Republic

* Correspondence: jan.zitko@faf.cuni.cz (J.Z.); Tel.: +420-495-067-272 (J.Z.)

Received: 29 August 2018; Accepted: 17 September 2018; Published: date

Supplementary Material

1. Full results of biological assays

1.1. Full results of antimycobacterial evaluation

Table S1. Antimycobacterial activity of prepared compounds expressed as MIC (μg/mL)

Cpd	R[1]	R[2]	*Mtb* H37Rv MIC (μg/mL)	*M. kans.* MIC (μg/mL)	*M. avium* MIC (μg/mL)	*M. smeg.* MIC (μg/mL)
**1a**	H	H	100	>100	>100	≥ 500
**1b**	H	2-OH	>100	>100	>100	≥ 500
**1c-Ac**	H	3-OAc	>100	>100	>100	≥ 500
**1c**	H	3-OH	>100	>100	>100	≥ 500
**1d-Ac**	H	4-OAc	>100	>100	>100	≥ 500
**1e**	H	2-OCH_3	>100	100	>100	125
**1f**	H	3-OCH_3	>100	>100	>100	≥ 500
**1g**	H	4-OCH_3	25	>100	>100	≥ 500
**1h**	H	4-CH_3	>100	>100	>100	≥ 500
**1i**	H	4-Et	>100	>100	>100	≥ 500
**1j**	H	2-Cl	>100	>100	>100	250
**1k**	H	3-Cl	50	50	>100	≥ 500
**1l**	H	4-Cl	50	50	>100	≥ 500
**1l-SP***	H	4-Cl*	50	>100	>100	≥ 500
**1n**	H	3-CF_3	>100	>100	>100	≥ 500
**2a**	5-Cl	H	>100	>100	>100	≥ 500
**2b-Ac**	5-Cl	2-OAc	12.5	12.5	50	31.25
**2b**	5-Cl	2-OH	12.5	50	50	15.625
**2c-Ac**	5-Cl	3-OAc	>100	>100	>100	250
**2c**	5-Cl	3-OH	>100	>100	>100	≥ 500
**2d-Ac**	5-Cl	4-OAc	>100	>100	>100	250
**2d**	5-Cl	4-OH	>100	>100	>100	≥ 500
**2e**	5-Cl	2-OCH_3	>100	>100	>100	≥ 500
**2f**	5-Cl	3-OCH_3	>100	>100	>100	≥ 500
**2g**	5-Cl	4-OCH_3	>100	>100	>100	≥ 500
**2h**	5-Cl	4-CH_3	6.25	>100	>100	≥ 500
**2i**	5-Cl	4-Et	3.13	>100	>100	≥ 500
**2j**	5-Cl	2-Cl	>100	>100	>100	≥ 500
**2k**	5-Cl	3-Cl	>100	>100	>100	≥ 500
**2l**	5-Cl	4-Cl	>100	>100	>100	≥ 500
**2m**	5-Cl	4-Br	>100	>100	>100	≥ 500
**2n**	5-Cl	3-CF_3	25	>100	>100	≥ 500
**3a**	6-Cl	H	>100 [a]	>100 [a]	>100 [a]	≥ 500 [a]
**3b**	6-Cl	2-OH	>100	>100	>100	250
**3e**	6-Cl	2-OCH_3	>100	>100	>100	≥ 500
**3f**	6-Cl	3-OCH_3	50 [a]	100 [a]	100 [a]	≥ 500 [a]
**3g**	6-Cl	4-OCH_3	>100	>100	>100	≥ 500 [a]
**3h**	6-Cl	4-CH_3	>100 [a]	>100 [a]	>100 [a]	≥ 500 [a]
**3i**	6-Cl	4-Et	>100 [a]	>100 [a]	>100 [a]	≥ 500 [a]
**3j**	6-Cl	2-Cl	>100	>100	>100	≥ 500
**3k**	6-Cl	3-Cl	>100 [a]	>100 [a]	>100 [a]	≥ 500 [a]
**3l**	6-Cl	4-Cl	>100 [a]	>100 [a]	>100 [a]	≥ 500 [a]
**3n**	6-Cl	3-CF_3	25	25	50	125
**INH**	-	-	0.1–0.39	6.25-12.5	6.25–12.5	15.625
**RFM**	-	-	-	-	-	1.56
**CPX**	-	-	-	-	-	0.195

* Diacylated side-product. [a] Precipitate formed in the testing medium. INH - isoniazid; RFM - rifampicin; CPX – ciprofloxacin.

1.2. Full results of antibacterial evaluation

Tested strains	
1. **SA** *Staphylococcus aureus* CCM 4516/08	
2. **MRSA** *Staphylococcus aureus* H 5996/08	**Methicillin** resistant
3. **SE** *Staphylococcus epidermidis* H 6966/08	
4. **EF** *Enterococcus sp.* J 14365/08	
5. **EC** *Escherichia coli* CCM4517	
6. **KP** *Klebsiella pneumoniae* D 11750/08	
7. **KP-E** *Klebsiella pneumoniae* J 14368/08	**ESBL** positive
8. **PA** *Pseudomonas aeruginosa* CCM 1961	

Results

**Strain**		**Tested compound** (lab code / publication code) – **MIC** (μM)								
		**OV-6**	**JZ-AM7**	**JZ-AM13**	**JZAM14**	**JZ-AM6**	**JZ-AM9**	**JZ-AM5**	**JZ-AM3**	**JZAM10**
		**1a**	**1b**	**1c-Ac**	**1c**	**1d-Ac**	**1e**	**1f**	**1g**	**1h**
**SA**	24 h	>500	>500	>500	>500	>500	>500	>500	>500	>500
	48 h	>500	>500	>500	>500	>500	>500	>500	>500	>500
**MRSA**	24 h	>500	>500	>500	>500	>500	>500	>500	>500	>500
	48 h	>500	>500	>500	>500	>500	>500	>500	>500	>500
**SE**	24 h	>500	>500	>500	>500	>500	>500	>500	>500	**500**
	48 h	>500	>500	>500	>500	>500	>500	>500	>500	**500**
**EF**	24 h	>500	>500	>500	>500	>500	>500	>500	>500	>500
	48 h	>500	>500	>500	>500	>500	>500	>500	>500	>500
**EC**	24 h	>500	>500	>500	>500	>500	>500	>500	>500	>500
	48 h	>500	>500	>500	>500	>500	>500	>500	>500	>500
**KP**	24 h	>500	>500	>500	>500	>500	>500	>500	>500	>500
	48 h	>500	>500	>500	>500	>500	>500	>500	>500	>500
**KP-E**	24 h	>500	>500	>500	>500	>500	>500	>500	>500	>500
	48 h	>500	>500	>500	>500	>500	>500	>500	>500	>500
**PA**	72 h	>500	>500	>500	>500	>500	>500	>500	>500	**500**
	120 h	>500	>500	>500	>500	>500	>500	>500	>500	**500**

Strain		Tested compound (lab code / publication code) – MIC (µM)								
		JZAM15	JZAM11	JZ-AM4	OV-5	JZ-AM2	JZ-AM8	OV-1	OV-14	OV-17
		1i	1j	1k	1l	1l-SP	1n	2a	2b-Ac	2b
**SA**	24 h	>500	**250**	>125	>125	>125	>125	**250**	**62.5**	**15.62**
	48 h	>500	**250**	>125	>125	>125	>125	**250**	**125**	**15.62**
**MRSA**	24 h	>500	>500	>125	>125	>125	>125	**250**	**125**	**62.5**
	48 h	>500	>500	>125	>125	>125	>125	**250**	**250**	**62.5**
**SE**	24 h	**500**	**500**	>125	>125	>125	**62.5**	**500**	**125**	**62.5**
	48 h	**500**	**500**	>125	>125	>125	**62.5**	**500**	**250**	**62.5**
**EF**	24 h	**500**	>500	>125	>125	>125	>125	>500	**250**	>500
	48 h	**500**	>500	>125	>125	>125	>125	>500	>250	>500
**EC**	24 h	>500	>500	>125	>125	>125	>125	>500	>250	>500
	48 h	>500	>500	>125	>125	>125	>125	>500	>250	>500
**KP**	24 h	>500	>500	>125	>125	>125	>125	>500	>250	**250**
	48 h	>500	>500	>125	>125	>125	>125	>500	>250	**250**
**KP-E**	24 h	>500	>500	>125	>125	>125	>125	>500	>250	**250**
	48 h	>500	>500	>125	>125	>125	>125	>500	>250	**250**
**PA**	72 h	>500	**250**	>125	>125	>125	>125	>500	>250	>500
	120 h	>500	**250**	>125	>125	>125	>125	>500	>250	>500

Strain		Tested compound (lab code / publication code) – MIC (µM)								
		OV-16	OV-19	OV-15	OV-18	OV-9	OV-7	OV-8	OV-11	OV-12
		2c-Ac	2c	2d-Ac	2d	2e	2f	2g	2h	2i
**SA**	24 h	>125	**500**	**0.98**	**31.25**	>125	**31.25**	>125	>125	>125
	48 h	>125	**500**	**0.98**	**31.25**	>125	**31.25**	>125	>125	>125
**MRSA**	24 h	>125	>500	>125	>500	>125	>500	>125	>125	>125
	48 h	>125	>500	>125	>500	>125	>500	>125	>125	>125
**SE**	24 h	>125	>500	>125	**500**	>125	>500	>125	>125	>125
	48 h	>125	>500	>125	**500**	>125	>500	>125	>125	>125
**EF**	24 h	>125	>500	>125	>500	>125	>500	>125	>125	>125
	48 h	>125	>500	>125	>500	>125	>500	>125	>125	>125
**EC**	24 h	>125	>500	>125	>500	>125	>500	>125	>125	>125
	48 h	>125	>500	>125	>500	>125	>500	>125	>125	>125
**KP**	24 h	>125	>500	>125	>500	>125	>500	>125	>125	>125
	48 h	>125	>500	>125	>500	>125	>500	>125	>125	>125
**KP-E**	24 h	>125	>500	>125	>500	>125	>500	>125	>125	>125
	48 h	>125	>500	>125	>500	>125	>500	>125	>125	>125
**PA**	72 h	>125	>500	>125	>500	>125	>500	>125	>125	>125
	120 h	>125	>500	>125	>500	>125	>500	>125	>125	>125

Strain		Tested compound (lab code / publication code) – MIC (µM)									
		OV-2A	OV-3	OV-4	OV-13	JZ-AM23	JZ-AM25	JZ-AM27			
		2j	2k	2l	2m	3e	3j	3k			
**SA**	24 h	**62.5**	>125	>125	>125	>500	>500	>125			
	48 h	**62.5**	>125	>125	>125	>500	>500	>125			
**MRSA**	24 h	>500	>125	>125	>125	>500	>500	>125			
	48 h	>500	>125	>125	>125	>500	>500	>125			
**SE**	24 h	>500	>125	>125	>125	>500	>500	>125			
	48 h	>500	>125	>125	>125	>500	>500	>125			
**EF**	24 h	>500	>125	>125	>125	>500	>500	>125			
	48 h	>500	>125	>125	>125	>500	>500	>125			
**EC**	24 h	>500	>125	>125	>125	>500	>500	>125			
	48 h	>500	>125	>125	>125	>500	>500	>125			
**KP**	24 h	>500	>125	>125	>125	>500	>500	>125			
	48 h	>500	>125	>125	>125	>500	>500	>125			
**KP-E**	24 h	>500	>125	>125	>125	>500	>500	>125			
	48 h	>500	>125	>125	>125	>500	>500	>125			
**PA**	72 h	>500	>125	>125	>125	>500	>500	>125			
	120 h	>500	>125	>125	>125	>500	>500	>125			

Compounds **1h**, **1i**, **2n**, **3a**, **3f**, **3g**, **3k**, **3l**, and **3n** precipitated during the preparation of the basic solution of the compound and therefore the testing was discontinued.

Strain		Tested standards – MIC (µM)									
		Neom	Bac	Pen	Phen	Cipr					
**SA**	24 h	**3.9**	**15.62**	**0.24**	**0.24**	**0.98**					
	48 h	**3.9**	**31.25**	**0.24**	**0.24**	**0.98**					
**MRSA**	24 h	**0.98**	**15.62**	**125**	**250**	**500**					
	48 h	**0.98**	**31.25**	**125**	**500**	**500**					
**SE**	24 h	**3.9**	**15.62**	**31.25**	**62.5**	**250**					
	48 h	**7.81**	**31.25**	**125**	**250**	**250**					
**EF**	24 h	**250**	**31.25**	**7.81**	**7.81**	**0.98**					
	48 h	**250**	**31.25**	**15.62**	**7.81**	**0.98**					
**EC**	24 h	**0.98**	>500	**125**	>500	**0.06**					
	48 h	**0.98**	>500	**125**	>500	**0.06**					
**KP**	24 h	**0.98**	>500	**250**	>500	**0.12**					
	48 h	**0.98**	>500	**500**	>500	**0.12**					
**KP-E**	24 h	**0.98**	>500	>500	>500	>500					
	48 h	**0.98**	>500	>500	>500	>500					
**PA**	72 h	**7.81**	>500	>500	>500	**3.9**					
	120 h	**15.62**	>500	>500	>500	**7.81**					

Neom – neomycin, Bac – bacitracin, Pen – benzylpenicillin, Phen – phenoxymethylpenicillin, Cipr – ciprofloxacin

1.3. Full results of antifungal evaluation

Tested strains	
1. **CA1** -*Candida albicans* ATCC 44859	5. **TA** -*Trichosporon asahii* 1188
2. **CT** -*Candida tropicalis* 156	6. **AF** -*Aspergillus fumigatus* 231
3. **CK2** -*Candida krusei* E28	7. **LC** -*Lichtheimia corymbifera* 272
4. **CG** -*Candida glabrata* 20/I	8. **TI** -*Trichophyton interdigitale* 445

Results

**Strain**		**Tested compound** (lab code / publication code) – **MIC** (µM)								
		**OV-6**	**JZ-AM7**	**JZ-AM13**	**JZAM14**	**JZ-AM6**	**JZ-AM9**	**JZ-AM5**	**JZ-AM3**	**JZAM10**
		**1a**	**1b**	**1c-Ac**	**1c**	**1d-Ac**	**1e**	**1f**	**1g**	**1h**
**CA**	24 h	**500**	>500	>500	>500	>500	>500	>500	>500	**500**
	48 h	>500	>500	>500	>500	>500	>500	>500	>500	**500**
**CT**	24 h	>500	>500	>500	>500	>500	>500	>500	>500	>500
	48 h	>500	>500	>500	>500	>500	>500	>500	>500	>500
**CK**	24 h	>500	>500	>500	>500	>500	>500	>500	>500	>500
	48 h	>500	>500	>500	>500	>500	>500	>500	>500	>500
**CG**	24 h	>500	>500	>500	>500	>500	>500	>500	>500	>500
	48 h	>500	>500	>500	>500	>500	>500	>500	>500	>500
**TA**	24 h	>500	>500	>500	>500	>500	>500	>500	>500	>500
	48 h	>500	>500	>500	>500	>500	>500	>500	>500	>500
**AF**	24 h	>500	>500	>500	>500	>500	>500	>500	>500	>500
	48 h	>500	>500	>500	>500	>500	>500	>500	>500	>500
**LC**	24 h	>500	>500	>500	>500	>500	>500	>500	>500	>500
	48 h	>500	>500	>500	>500	>500	>500	>500	>500	>500
**TI**	72 h	>500	**500**	>500	>500	>500	>500	>500	>500	>500
	120 h	>500	**500**	>500	>500	>500	>500	>500	>500	>500

Strain		Tested compound (lab code / publication code) – MIC (µM)									
		JZAM115	JZAM11	JZ-AM4	OV-5	JZ-AM2	JZ-AM8	OV-1	OV-14	OV-17	
		1i	1j	1k	1l	1l-SP	1n	2a	2b-Ac	2b	
**CA**	24 h	>500	>500	>125	>125	>125	>125	>500	**125**	**62.5**	
	48 h	>500	>500	>125	>125	>125	>125	>500	**125**	**62.5**	
**CT**	24 h	>500	>500	>125	>125	>125	>125	>500	**250**	**125**	
	48 h	>500	>500	>125	>125	>125	>125	>500	**250**	**125**	
**CK**	24 h	>500	>500	>125	>125	>125	>125	>500	**125**	**125**	
	48 h	>500	>500	>125	>125	>125	>125	>500	**125**	**125**	
**CG**	24 h	>500	>500	>125	>125	>125	>125	>500	**250**	**125**	
	48 h	>500	>500	>125	>125	>125	>125	>500	**250**	**125**	
**TA**	24 h	>500	>500	>125	>125	>125	>125	>500	**125**	**62.5**	
	48 h	>500	>500	>125	>125	>125	>125	>500	**125**	**125**	
**AF**	24 h	>500	>500	>125	>125	>125	>125	>500	**250**	**125**	
	48 h	>500	>500	>125	>125	>125	>125	>500	**250**	**125**	
**LC**	24 h	>500	>500	>125	>125	>125	>125	>500	**125**	**62.5**	
	48 h	>500	>500	>125	>125	>125	>125	>500	**125**	**125**	
**TI**	72 h	>500	>500	>125	>125	>125	>125	>500	**250**	**125**	
	120 h	>500	>500	>125	>125	>125	>125	>500	**250**	**125**	

Strain		Tested compound (lab code / publication code) – MIC (µM)									
		OV-16	OV-19	OV-15	OV-18	OV-9	OV-7	OV-8	OV-11	OV-12	
		2c-Ac	2c	2d-Ac	2d	2e	2f	2g	2h	2i	
**CA**	24 h	>125	**500**	>125	**500**	>125	**125**	>125	>125	>125	
	48 h	>125	>500	>125	>500	>125	**500**	>125	>125	>125	
**CT**	24 h	>125	>500	>125	>500	>125	>500	>125	>125	>125	
	48 h	>125	>500	>125	>500	>125	>500	>125	>125	>125	
**CK**	24 h	>125	>500	>125	>500	>125	>500	>125	>125	>125	
	48 h	>125	>500	>125	>500	>125	>500	>125	>125	>125	
**CG**	24 h	>125	>500	>125	>500	>125	>500	>125	>125	>125	
	48 h	>125	>500	>125	>500	>125	>500	>125	>125	>125	
**TA**	24 h	>125	>500	>125	>500	>125	>500	>125	>125	>125	
	48 h	>125	>500	>125	>500	>125	>500	>125	>125	>125	
**AF**	24 h	>125	>500	>125	>500	>125	>500	>125	>125	>125	
	48 h	>125	>500	>125	>500	>125	>500	>125	>125	>125	
**LC**	24 h	>125	>500	>125	>500	>125	>500	>125	>125	>125	
	48 h	>125	>500	>125	>500	>125	>500	>125	>125	>125	
**TI**	72 h	>125	>500	>125	>500	>125	>500	>125	>125	>125	
	120 h	>125	>500	>125	>500	>125	>500	>125	>125	>125	

Strain		**Tested compound** (lab code / publication code) – **MIC** (µM)									
		**OV-2A**	**OV-3**	**OV-4**	**OV-13**	**JZ-AM23**	**JZ-AM25**	**JZ-AM27**			
		**2j**	**2k**	**2l**	**2m**	**3e**	**3j**	**3k**			
**CA**	24 h	>500	>125	>125	>125	>500	>500	>125			
	48 h	>500	>125	>125	>125	>500	>500	>125			
**CT**	24 h	>500	>125	>125	>125	>500	>500	>125			
	48 h	>500	>125	>125	>125	>500	>500	>125			
**CK**	24 h	>500	>125	>125	>125	>500	>500	>125			
	48 h	>500	>125	>125	>125	>500	>500	>125			
**CG**	24 h	>500	>125	>125	>125	>500	>500	>125			
	48 h	>500	>125	>125	>125	>500	>500	>125			
**TA**	24 h	>500	>125	>125	>125	>500	>500	>125			
	48 h	>500	>125	>125	>125	>500	>500	>125			
**AF**	24 h	>500	>125	>125	>125	>500	>500	>125			
	48 h	>500	>125	>125	>125	>500	>500	>125			
**LC**	24 h	>500	>125	>125	>125	>500	>500	>125			
	48 h	>500	>125	>125	>125	>500	>500	>125			
**TI**	72 h	>500	>125	>125	>125	>500	>500	>125			
	120 h	>500	>125	>125	>125	>500	>500	>125			

Compounds **1h**, **1i**, **2n**, **3a**, **3f**, **3g**, **3k**, **3l**, and **3n** precipitated during the preparation of the basic solution of the compound and therefore the testing was discontinued.

Strain		**Tested standard drugs** (lab code) – **MIC** (µM)									
		**AMPB**	**NYS**	**FLU**	**VOR**						
**CA**	24 h	**0.12**	**0.98**	**0.24**	**0.002**						
	48 h	**0.49**	**1.95**	**0.24**	**0.008**						
**CT**	24 h	**1.95**	**1.95**	>500	**62.5**						
	48 h	**1.95**	**3.9**	>500	**250**						
**CK**	24 h	**1.95**	**1.95**	**125**	**0.49**						
	48 h	**1.95**	**3.9**	**250**	**1.95**						
**CG**	24 h	**0.98**	**1.95**	**31.25**	**0.24**						
	48 h	**1.95**	**3.9**	**250**	**250**						
**TA**	24 h	**0.49**	**1.95**	**250**	**0.98**						
	48 h	**0.98**	**1.95**	**500**	**7.81**						
**AF**	24 h	**1.95**	**1.95**	>500	**0.49**						
	48 h	**1.95**	**3.9**	>500	**1.95**						
**LC**	24 h	**7.81**	**15.62**	>500	**125**						
	48 h	**7.81**	**31.25**	>500	**250**						
**TI**	72 h	**1.95**	**3.9**	**7.81**	**0.061**						
	120 h	**1.95**	**7.81**	**125**	**0.12**						

AMPB - amphotericin B, NYS – nystatin, FLU – fluconazole, VOR – voriconazole

2. Experimental

2.1. Analytical data of prepared compounds

In the following interpretation of ^{1}H NMR spectra, the hydrogens of the benzene core are annotated with non-primed numbers (*e.g.* H2) or non-specifically as ArH. Hydrogens of the pyrazine are annotated with primed numbers (*e.g.* H3′).

N-(pyrazin-2-yl)benzamide (**1a**). White solid. Yield: 59%. mp 171.2–172.0 °C. ^{1}H NMR (500 MHz, $CDCl_3$) δ 9.73 (d, *J* = 1.6 Hz, 1H, H3′), 8.78 (bs, 1H, NH), 8.37 (bs, 1H, H5′), 8.22 (bs, 1H, H6′), 7.94 (d, *J* = 7.5 Hz, 2H, H2, H6), 7.63–7.56 (m, 1H, H4), 7.54–7.48 (m, 2H, H3, H5). ^{13}C NMR (126 MHz, $CDCl_3$) δ 165.50, 148.29, 141.87, 140.17, 137.20, 133.32, 132.69, 128.93, 127.37. IR (ATR-Ge, cm^{-1}): 3234; 3108; 3063; 2920; 2851; 1676 (C=O, amide); 1531; 1409; 1294; 1263; 1151; 1057; 1014; 844; 802; 705. Anal. calcd. for $C_{11}H_9N_3O$ (MW 199.21): C, 66.32; H, 4.55; N, 21.09. Found: C, 66.11; H, 4.57; N, 20.83.

2-hydroxy-*N*-(pyrazin-2-yl)benzamide (**1b**). White solid. Yield: 67%. mp 209.9–211.5 °C (lit. 213–216 °C [1]). ^{1}H NMR (500 MHz, DMSO-d_6) δ 11.77 (bs, 1H, OH), 11.01 (s, 1H, NH), 9.51 (d, *J* = 1.4 Hz, 1H, H3′), 8.43 (dd, *J* = 2.5, 1.5 Hz, 1H, H6′), 8.42 (d, *J* = 2.6 Hz, 1H, H5′), 8.03 (dd, *J* = 8.0, 1.8 Hz, 1H, H6), 7.49–7.44 (m, 1H, H4), 7.06 (dd, *J* = 8.2, 1.1 Hz, 1H, H3), 7.03–6.98 (m, 1H, H5). ^{13}C NMR (126 MHz, DMSO-d_6) δ 164.47, 156.91, 148.52, 142.99, 140.37, 136.82, 134.46, 130.96, 120.04, 117.84, 117.31. IR (ATR-Ge, cm^{-1}): 3274; 2933; 2566; 1669 (C=O, amide); 1538; 1449; 1417; 1301; 1222; 1149; 1063; 1012; 839; 759; 689. Anal. calcd. for $C_{11}H_9N_3O_2$ (MW 215.21): C, 61.39; H, 4.22; N, 19.53. Found: C, 61.24; H, 4.12; N, 19.13.

3-(pyrazin-2-ylcarbamoyl)phenyl acetate (**1c-Ac**). White solid. Yield: 60%. mp 168.4–169.5 °C. ^{1}H NMR (500 MHz, $CDCl_3$) δ 9.69 (s, 1H, H3′), 8.63 (s, 1H, NH), 8.38 (d, *J* = 2.6 Hz, 1H, H5′), 8.25 (s, 1H, H6′), 7.78 (d, *J* = 7.8 Hz, 1H, H6), 7.68 (s, 1H, H2), 7.52 (t, *J* = 7.9 Hz, 1H, H5), 7.33 (d, *J* = 7.7 Hz, 1H, H4), 2.33 (s, 3H, CH_3). ^{13}C NMR (126 MHz, $CDCl_3$) δ 169.12, 164.41, 151.03, 148.11, 142.08, 140.52, 137.22, 134.90, 130.02, 125.98, 124.51, 121.01, 21.04. IR (ATR-Ge, cm^{-1}): 3244; 1768 (C=O, ester); 1683 (C=O, amide); 1541; 1421; 1304; 1271; 1215; 1194; 1016; 845; 716; 676. Anal. calcd. for $C_{13}H_{11}N_3O_3$ (MW 257.25): C, 60.7; H, 4.31; N, 16.33. Found: C, 60.67; H, 4.22; N, 16.11.

3-hydroxy-*N*-(pyrazin-2-yl)benzamide (**1c**). White solid. Yield: 58%. mp 192.2–193.7 °C. ^{1}H NMR (500 MHz, DMSO-d_6) δ 10.98 (bs, 1H, NH), 9.78 (bs, 1H,OH), 9.39 (d, *J* = 1.5 Hz, 1H, H3′), 8.46 (dd, *J* = 2.6, 1.5 Hz, 1H, H6′), 8.40 (d, *J* = 2.5 Hz, 1H, H5′), 7.52–7.47 (m, 1H, ArH), 7.40 (t, *J* = 2.1 Hz, 1H, ArH), 7.32 (t, *J* = 7.8 Hz, 1H, ArH), 7.03–6.97 (m, 1H, ArH). ^{13}C NMR (126 MHz, DMSO-d_6) δ 166.37, 157.56, 149.24, 142.75, 140.07, 137.72, 134.98, 129.65, 119.45, 118.87, 115.17. IR (ATR-Ge, cm^{-1}): 3343; 3158; 2851; 2705; 1679 (C=O, amide); 1539; 1515; 1414; 1297; 1253; 1012; 819; 749; 691. Anal. calcd. for $C_{11}H_9N_3O_2$ (MW 215.21): C, 61.39; H, 4.22; N, 19.53. Found: C, 61.19; H, 4.23; N, 19.12.

4-(pyrazin-2-ylcarbamoyl)phenyl acetate (**1d-Ac**). White solid. Yield: 14%. mp 147.4–149.4 °C (lit. 158 °C [2]). ^{1}H NMR (500 MHz, $CDCl_3$) δ 9.70 (d, *J* = 1.7 Hz, 1H, H3′), 8.70 (bs, 1H, NH), 8.37 (d, *J* = 2.6 Hz, 1H, H5′), 8.22 (bs, 1H, H6′), 7.96 (d, *J* = 8.5 Hz, 2H, H2, H6), 7.24 (d, *J* = 8.5 Hz, 2H, H3, H5), 2.33 (s, 3H, CH_3). ^{13}C NMR (126 MHz, $CDCl_3$) δ 168.82, 164.60, 153.95, 148.22, 142.02, 140.41, 137.24, 130.86, 128.90, 122.16, 21.10. IR (ATR-Ge, cm^{-1}): 3171; 3107; 3046; 1753 (C=O, ester; 1672

(C=O, amide); 1541; 1504; 1417; 1300; 1209; 1166; 1015; 916; 843; 757; 704. Anal. calcd. for $C_{13}H_{11}N_3O_3$ (MW 257.25): C, 60.7; H, 4.31; N, 16.33. Found: C, 60.23; H, 4.17; N, 15.98.

2-methoxy-*N*-(pyrazin-2-yl)benzamide (**1e**). White solid. Yield: 11%. mp 100.3–102.4 °C. ^{1}H NMR (500 MHz, $CDCl_3$) δ 10.37 (bs, 1H, NH), 9.74 (d, *J* = 1.5 Hz, 1H, H3′), 8.33 (d, *J* = 2.5 Hz, 1H, H5′), 8.30–8.25 (m, 2H, H6′, H6), 7.56–7.50 (m, 1H, H4), 7.16–7.11 (m, 1H, H5), 7.05 (d, *J* = 8.4 Hz, 1H, H3), 4.08 (s, 3H, OCH_3). ^{13}C NMR (126 MHz, $CDCl_3$) δ 163.36, 157.52, 148.67, 142.09, 139.96, 137.67, 134.12, 132.60, 121.63, 120.49, 111.58, 56.22. IR (ATR-Ge, cm^{-1}): 3335; 3016; 2955; 1673 (C=O, amide); 1533; 1483; 1414; 1297; 1234; 1016; 844; 745; 685; 669. Anal. calcd. for $C_{12}H_{11}N_3O_2$ (MW 229.24): C, 62.87; H, 4.84; N, 18.33. Found: C, 62.82; H, 4.8; N, 17.91.

3-methoxy-*N*-(pyrazin-2-yl)benzamide (**1f**). White solid. Yield: 22%. mp 148.1–150.1 °C. ^{1}H NMR (500 MHz, $CDCl_3$) δ 9.71 (d, *J* = 1.5 Hz, 1H, H3′), 8.71 (bs, 1H, NH), 8.36 (d, *J* = 2.6 Hz, 1H, H5′), 8.24–8.20 (m, 1H, H6′), 7.50–7.43 (m, 2H, ArH), 7.40 (t, *J* = 7.9 Hz, 1H, ArH), 7.12 (dd, *J* = 8.7, 2.7 Hz, 1H, ArH), 3.86 (s, 3H, OCH_3). ^{13}C NMR (126 MHz, $CDCl_3$) δ 165.35, 160.04, 148.26, 142.02, 140.33, 137.24, 134.79, 129.90, 119.05, 118.88, 112.64, 55.48. IR (ATR-Ge, cm^{-1}): 3171; 3110; 3003; 2976; 1673 (C=O, amide); 1544; 1417; 1300; 1277; 1235; 1033; 1017; 797; 740. Anal. calcd. for $C_{12}H_{11}N_3O_2$ (MW 229.24): C, 62.87; H, 4.84; N, 18.33. Found: C, 63.08; H, 4.83; N, 18.01.

4-methoxy-*N*-(pyrazin-2-yl)benzamide (**1g**). White solid. Yield: 14%. mp 156.0–156.6 °C (lit. 157–159 [3]). ^{1}H NMR (300 MHz, $CDCl_3$) δ 9.70 (d, *J* = 1.5 Hz, 1H, H3′), 8.68 (bs, 1H, NH), 8.34 (d, *J* = 2.5 Hz, 1H, H5′), 8.23–8.19 (m, 1H, H6′), 7.90 (d, *J* = 8.4 Hz, 2H, H2. H6), 6.98 (d, *J* = 8.4 Hz, 2H, H3, H5), 3.87 (s, 3H, OCH_3). ^{13}C NMR (75 MHz, $CDCl_3$) δ 164.95, 163.09, 148.49, 141.94, 140.02, 137.24, 129.36, 125.42, 114.10, 55.48. IR (ATR-Ge, cm^{-1}): 3304; 3116; 2927; 2844; 1663 (C=O, amide); 1508; 1414; 1299; 1249; 1173; 1026; 1012; 835; 759; 656. Anal. calcd. for $C_{12}H_{11}N_3O_2$ (MW 229.24): C, 62.87; H, 4.84; N, 18.33. Found: C, 62.58; H, 4.83; N, 17.95.

4-methyl-*N*-(pyrazin-2-yl)benzamide (**1h**). White solid. Yield: 29%. mp 163.3–166.0 °C (lit. [4]). ^{1}H NMR (500 MHz, $CDCl_3$) δ 9.71 (d, *J* = 1.7 Hz, 1H, H3′), 8.66 (bs, 1H, NH), 8.35 (d, *J* = 2.6 Hz, 1H, H5′), 8.23 (dd, *J* = 2.5, 1.6 Hz, 1H, H6′), 7.83 (d, *J* = 8.2 Hz, 2H, H2, H6), 7.30 (d, *J* = 7.9 Hz, 2H, H3, H5), 2.43 (s, 3H, CH_3). ^{13}C NMR (126 MHz, $CDCl_3$) δ 165.39, 148.40, 143.42, 141.97, 140.15, 137.23, 130.47, 129.59, 127.37, 21.54. IR (ATR-Ge, cm^{-1}): 3245; 3109; 3063; 1676 (C=O, amide); 1529; 1508; 1412; 1298; 1259; 1186; 1057; 1012; 847; 723; 685. Anal. calcd. for $C_{12}H_{11}N_3O$ (MW 213.24): C, 67.59; H, 5.2; N, 19.71. Found: C, 67.37; H, 5.1; N, 19.59.

4-ethyl-*N*-(pyrazin-2-yl)benzamide (**1i**). White solid. Yield: 15%. mp 121.2–122.3 °C. ^{1}H NMR (500 MHz, $CDCl_3$) δ 9.72 (d, *J* = 1.5 Hz, 1H, H3′), 8.67 (bs, 1H, NH), 8.35 (d, *J* = 2.6 Hz, 1H, H5′), 8.22 (dd, *J* = 2.6, 1.6 Hz, 1H, H6′), 7.85 (d, *J* = 8.3 Hz, 2H, H2, H6), 7.33 (d, *J* = 8.2 Hz, 2H, H3, H5), 2.72 (q, *J* = 7.6 Hz, 2H, CH_2), 1.27 (t, *J* = 7.6 Hz, 3H, CH_3). ^{13}C NMR (126 MHz, $CDCl_3$) δ 165.42, 149.58, 148.41, 141.98, 140.17, 137.25, 130.70, 128.41, 127.47, 28.82, 15.17. IR (ATR-Ge, cm^{-1}): 3239; 3107; 3049; 2959; 2933; 2875; 1685 (C=O, amide); 1534; 1509; 1411; 1299; 1269; 1014; 840; 734; 695. Anal. calcd. for $C_{13}H_{13}N_3O$ (MW 227.27): C, 68.7; H, 5.77; N, 18.49. Found: C, 68.89; H, 5.72; N, 18.4.

2-chloro-*N*-(pyrazin-2-yl)benzamide (**1j**). White solid. Yield: 15%. mp 128.2–129.7 °C (lit. 136–138 °C [4]). ^{1}H NMR (500 MHz, $CDCl_3$) δ 9.68 (s, 1H, H3′), 9.07 (bs, 1H, NH), 8.34 (d, *J* = 2.6 Hz, 1H, H5′), 8.05 (dd, *J* = 2.6, 1.6 Hz, 1H, H6′), 7.78–7.74 (m, 1H, ArH), 7.47–7.45 (m, 2H, ArH), 7.42–7.37 (m, 1H ,ArH). ^{13}C NMR (126 MHz, $CDCl_3$) δ 164.65, 147.93, 141.91, 140.62, 137.29, 134.06, 132.31,

130.93, 130.58, 130.28, 127.37. IR (ATR-Ge, cm^{-1}): 3306; 3198; 3100; 2971; 1695 (C=O, amide); 1530; 1412; 1299; 1253; 1151; 1048; 1015; 852; 774; 721. Anal. calcd. for $C_{11}H_8ClN_3O$ (MW 233.66): C, 56.55; H, 3.45; N, 17.98. Found: C, 56.86; H, 3.45; N, 17.51.

3-chloro-*N*-(pyrazin-2-yl)benzamide (**1k**). White solid. Yield: 35%. mp 147.2–149.4 °C. ^{1}H NMR (500 MHz, $CDCl_3$) δ 9.69 (d, *J* = 1.7 Hz, 1H, H3′), 8.71 (bs, 1H, NH), 8.38 (d, *J* = 2.6 Hz, 1H, H5′), 8.25 (bs, 1H, H6′), 7.93 (s, 1H, H2), 7.80 (d, *J* = 7.7 Hz, 1H, H6), 7.56 (d, *J* = 7.8 Hz, 1H, H4), 7.45 (t, *J* = 7.9 Hz, 1H, H5). ^{13}C NMR (126 MHz, $CDCl_3$) δ 164.18, 148.04, 142.08, 140.62, 137.26, 135.21, 135.14, 132.66, 130.22, 127.78, 125.33. IR (ATR-Ge, cm^{-1}): 3178; 3107; 3069; 1680 (C=O, amide); 1545; 1414; 1305; 1262; 1058; 1018; 846; 765; 725. Anal. calcd. for $C_{11}H_8ClN_3O$ (MW 233.66): C, 56.55; H, 3.45; N, 17.98. Found: C, 56.16; H, 3.44; N, 17.87.

4-chloro-*N*-(pyrazin-2-yl)benzamide (**1l**). White solid. Yield: 11%. mp 182.6–184.3 °C. ^{1}H NMR (500 MHz, $CDCl_3$) δ 9.71 (s, 1H, H3′), 8.69 (bs, 1H, NH), 8.40 (d, *J* = 2.8 Hz, 1H, H5′), 8.27 (s, 1H, H6′), 7.89 (d, *J* = 8.3 Hz, 2H, H2, H6), 7.49 (d, *J* = 8.2 Hz, 2H, H3, H5). ^{13}C NMR (126 MHz, $CDCl_3$) δ 164.38, 148.02, 141.70, 140.30, 139.22, 137.21, 131.58, 129.28, 128.82. IR (ATR-Ge, cm^{-1}): 3330; 3067; 1655 (C=O, amide); 1540; 1488; 1408; 1302; 1262; 1149; 1009; 846; 754; 662. Anal. calcd. for $C_{11}H_8ClN_3O$ (MW 233.66): C, 56.55; H, 3.45; N, 17.98. Found: C, 56.48; H, 3.43; N, 17.7.

4-chloro-*N*-(4-chlorobenzoyl)-*N*-(pyrazin-2-yl)benzamide (**1l-SP**). White solid. Yield: 61%. mp 153.1–153.8 °C. ^{1}H NMR (500 MHz, $CDCl_3$) δ 8.63 (d, *J* = 1.5 Hz, 1H, H3′), 8.45 (d, *J* = 2.6 Hz, 1H, H5′), 8.35 (dd, *J* = 2.4, 1.5 Hz, 1H, H6′), 7.72–7.63 (m, 4H, ArH), 7.38–7.33 (m, 4H, ArH). IR (ATR-Ge, cm^{-1}): 1703 (C=O, amide, široký); 1590; 1403; 1269; 1240; 1113; 1089; 1011; 920; 872; 845; 754. Anal. calcd. for $C_{18}H_{11}Cl_2N_3O_2$ (MW 372.21): C, 58.09; H, 2.98; N, 11.29. Found: C, 58.5; H, 2.91; N, 11.15.

N-(pyrazin-2-yl)-3-(trifluoromethyl)benzamide (**1n**). White solid. Yield: 21%. mp 160.9–161.6 °C (lit. 159–161 °C [5]). ^{1}H NMR (500 MHz, $CDCl_3$) δ 9.70 (d, *J* = 1.6 Hz, 1H, H3′), 8.80 (bs, 1H, NH), 8.40 (d, *J* = 2.6 Hz, 1H, H5′), 8.28–8.25 (m, 1H, H6′), 8.21 (s, 1H, H2), 8.13 (d, *J* = 7.8 Hz, 1H, ArH), 7.85 (d, *J* = 7.7 Hz, 1H, ArH), 7.67 (t, *J* = 7.8 Hz, 1H, H5). ^{13}C NMR (126 MHz, $CDCl_3$) δ 164.10, 147.98, 142.11, 140.74, 137.29, 134.26, 131.59 (q, *J* = 33.1 Hz), 130.54, 129.63, 129.19 (q, *J* = 3.6 Hz), 124.49 (q, *J* = 3.8 Hz), 123.46 (q, *J* = 272.7 Hz). IR (ATR-Ge, cm^{-1}): 3177; 3107; 1681 (C=O, amide); 1548; 1419; 1337; 1305; 1257; 1167; 1125; 1074; 1016; 740; 694. Anal. calcd. for $C_{12}H_8F_3N_3O$ (MW 267.21): C, 53.94; H, 3.02; N, 15.73. Found: C, 54.02; H, 2.83; N, 15.49.

N-(5-chloropyrazin-2-yl)benzamide (**2a**). White solid. Yield: 48%. mp 162.5–163.5 °C. ^{1}H NMR (500 MHz, DMSO-d_6) δ 11.31 (s, 1H, NH), 9.26 (d, *J* = 1.4 Hz, 1H, H3′), 8.62 (d, *J* = 1.5 Hz, 1H, H6′), 8.08–8.01 (m, 2H, H2, H6), 7.66–7.59 (m, 1H, H4), 7.56–7.50 (m, 2H, H3, H5). ^{13}C NMR (126 MHz, DMSO-d_6) δ 166.37, 148.26, 142.26, 141.96, 136.46, 133.32, 132.58, 128.61, 128.40. IR (ATR-Ge, cm^{-1}): 3342; 1655 (C=O, amide); 1541; 1511; 1484; 1340; 1249; 1131; 1103; 1020; 710; 690; 661. Anal. calcd. for $C_{11}H_8ClN_3O$ (MW 233.66): C, 56.55; H, 3.45; N, 17.98. Found: C, 56.89; H, 3.23; N, 18.22.

N-(5-chloropyrazin-2-yl)-2-hydroxybenzamide (**2b**). White solid. Yield: 37%. mp 197.7–198.2 °C. ^{1}H NMR (500 MHz, DMSO-d_6) δ 11.78 (s, 1H, OH), 11.12 (s, 1H, NH), 9.31 (d, *J* = 1.6 Hz, 1H, H3′), 8.56 (d, *J* = 1.4 Hz, 1H, H6′), 8.01 (dd, *J* = 8.0, 1.7 Hz, 1H, H6), 7.51–7.42 (m, 1H, H4), 7.05 (d, *J* = 8.2 Hz, 1H, H3), 7.00 (t, *J* = 7.4 Hz, 1H, H5). ^{13}C NMR (126 MHz, DMSO-d_6) δ 164.40, 156.85, 147.47, 142.49, 141.99, 135.59, 134.56, 130.93, 120.05, 117.68, 117.29. IR (ATR-Ge, cm^{-1}): 3383; 3034; 1651

(C=O, amide); 1538; 1515; 1490; 1433; 1339; 1197; 1170; 1136; 1013; 819; 751; 708. Anal. calcd. for $C_{11}H_8ClN_3O_2$ (MW 249.65): C, 52.92; H, 3.23; N, 16.83. Found: C, 53.2; H, 3.36; N, 16.855.

2-((5-chloropyrazin-2-yl)carbamoyl)phenyl acetate (**2b**-**Ac**). White solid. Yield: 53%. mp 116.3–117.0 °C. ^{1}H NMR (500 MHz, DMSO-*d*$_6$) δ 11.35 (s, 1H, NH), 9.18 (d, *J* = 1.5 Hz, 1H, H3′), 8.61 (d, *J* = 1.4 Hz, 1H, H6′), 7.75 (dd, *J* = 7.7, 1.6 Hz, 1H, ArH), 7.65–7.57 (m, 1H, ArH), 7.42–7.38 (m, 1H, ArH), 7.27 (dd, *J* = 8.1, 1.1 Hz, 1H, ArH), 2.22 (s, 3H, CH_3). ^{13}C NMR (126 MHz, DMSO-*d*$_6$) δ 169.02, 165.09, 148.41, 147.90, 142.45, 142.11, 135.98, 132.51, 129.77, 128.09, 125.96, 123.45, 20.87. IR (ATR-Ge, cm^{-1}): 3404; 1766 (C=O, ester); 1685 (C=O, amide); 1534; 1501; 1343; 1192; 1159; 1136; 1017; 916; 748; 682. Anal. calcd. for $C_{13}H_{10}ClN_3O_3$ (MW 291.69): C, 53.53; H, 3.46; N, 14.41. Found: C, 53.385; H, 3.38; N, 14.39.

N-(5-chloropyrazin-2-yl)-3-hydroxybenzamide (**2c**). White solid. Yield: 48%. mp 190.1–190.8 °C. ^{1}H NMR (500 MHz, DMSO-*d*$_6$) δ 11.19 (s, 1H, NH), 9.79 (s, 1H, OH), 9.23 (d, *J* = 1.4 Hz, 1H, H3′), 8.61 (d, *J* = 1.4 Hz, 1H, H6′), 7.50–7.45 (m, 1H, ArH), 7.39 (t, *J* = 2.0 Hz, 1H, ArH), 7.32 (t, *J* = 7.9 Hz, 1H, ArH), 7.03–6.99 (m, 1H, ArH). ^{13}C NMR (126 MHz, DMSO-*d*$_6$) δ 166.40, 157.56, 148.25, 142.25, 141.92, 136.50, 134.72, 129.68, 119.58, 118.93, 115.19. IR (ATR-Ge, cm^{-1}): 3322; 1651 (C=O, amide); 1595; 1540; 1494; 1446; 1340; 1296; 1231; 1135; 1030; 827; 804; 750; 712; 684. Anal. calcd. for $C_{11}H_8ClN_3O_2$ (MW 249.65): C, 52.92; H, 3.23; N, 16.83. Found: C, 53.15; H, 3.235; N, 16.885.

3-((5-chloropyrazin-2-yl)carbamoyl)phenyl acetate (**2c**-**Ac**). White solid. Yield: 48%. mp 184.1–184.6 °C. ^{1}H NMR (500 MHz, DMSO-*d*$_6$) δ 11.38 (s, 1H, NH), 9.24 (d, *J* = 1.5 Hz, 1H, H3′), 8.63 (d, *J* = 1.4 Hz, 1H, H6′), 7.95 (d, *J* = 7.8 Hz, 1H, H6), 7.82 (t, *J* = 2.0 Hz, 1H, H2), 7.58 (t, *J* = 7.9 Hz, 1H, H5), 7.43–7.38 (m, 1H, H4), 2.31 (s, 3H, CH_3). ^{13}C NMR (126 MHz, DMSO-*d*$_6$) δ 169.31, 165.36, 150.60, 148.10, 142.32, 142.13, 136.51, 134.70, 129.83, 126.18, 125.81, 121.95, 21.00. IR (ATR-Ge, cm^{-1}): 3357; 3126; 3080; 1758 (C=O, ester); 1668 (C=O, amide); 1542; 1510; 1483; 1351; 1211; 1135; 1020; 822; 746; 690; 661. Anal. calcd. for $C_{13}H_{10}ClN_3O_3$ (MW 291.69): C, 53.53; H, 3.46; N, 14.41. Found: C, 53.55; H, 3.55; N, 14.455.

N-(5-chloropyrazin-2-yl)-4-hydroxybenzamide (**2d**). White solid. Yield: 36%. mp 210.9–212.6 °C. ^{1}H NMR (500 MHz, DMSO-*d*$_6$) δ 11.02 (s, 1H, NH), 10.26 (bs, 1H, OH), 9.23 (s, 1H, H3′), 8.59 (s, 1H, H6′), 7.95 (d, *J* = 8.4 Hz, 2H, H2, H6), 6.86 (d, *J* = 8.4 Hz, 2H, H3, H5). ^{13}C NMR (126 MHz, DMSO-*d*$_6$) δ 165.77, 161.57, 148.55, 142.12, 141.53, 136.39, 130.69, 123.68, 115.22. IR (ATR-Ge, cm^{-1}): 3106; 1644 (C=O, amide); 1588; 1532; 1505; 1434; 1336; 1270; 1235; 1139; 1018; 911; 843; 738; 710. Anal. calcd. for $C_{11}H_8ClN_3O_2$ (MW 249.65): C, 52.92; H, 3.23; N, 16.83. Found: C, 53.025; H, 3.445; N, 16.77.

4-((5-chloropyrazin-2-yl)carbamoyl)phenyl acetate (**2d**-**Ac**). White solid. Yield: 55%. mp 170.9–171.8 °C. ^{1}H NMR (500 MHz, DMSO-*d*$_6$) δ 11.36 (s, 1H, NH), 9.25 (d, *J* = 1.4 Hz, 1H, H3′), 8.63 (d, *J* = 1.5 Hz, 1H, H6′), 8.09 (d, *J* = 8.7 Hz, 2H, H2, H6), 7.30 (d, *J* = 8.7 Hz, 2H, H3, H5), 2.31 (s, 3H, CH_3). ^{13}C NMR (126 MHz, DMSO-*d*$_6$) δ 169.07, 165.60, 153.73, 148.23, 142.27, 141.99, 136.42, 130.83, 130.05, 122.09, 21.07. IR (ATR-Ge, cm^{-1}): 3396; 3090; 1761 (C=O, ester); 1683 (C=O, amide); 1535; 1497; 1348; 1171; 1136; 1016; 911; 854; 755. Anal. calcd. for $C_{13}H_{10}ClN_3O_3$ (MW 291.69): C, 53.53; H, 3.46; N, 14.41. Found: C, 53.92; H, 3.68; N, 14.23.

N-(5-chloropyrazin-2-yl)-2-methoxybenzamide (**2e**). White solid. Yield: 75%. mp 141.2–142.0 °C. ^{1}H NMR (300 MHz, DMSO-*d*$_6$) δ 10.80 (s, 1H, NH), 9.30 (d, *J* = 1.4 Hz, 1H, H3′), 8.57 (d, *J* = 1.4 Hz, 1H, H6′), 7.83 (dd, *J* = 7.6, 1.8 Hz, 1H, ArH), 7.65–7.51 (m, 1H, ArH), 7.24 (d, *J* = 8.4 Hz, 1H, ArH), 7.15–7.07 (m, 1H, ArH), 3.96 (s, 3H, OCH_3). ^{13}C NMR (75 MHz, DMSO-*d*$_6$) δ 164.47, 157.26, 147.55,

142.49, 141.99, 135.46, 133.82, 130.75, 121.96, 121.05, 112.58, 56.45. IR (ATR-Ge, cm^{-1}): 3309; 1677 (C=O, amide); 1601; 1531; 1511; 1484; 1344; 1293; 1227; 1116; 1013; 748; 680. Anal. calcd. for $C_{12}H_{10}ClN_3O_2$ (MW 263.68): C, 54.66; H, 3.82; N, 15.94. Found: C, 54.48; H, 3.77; N, 15.75.

N-(5-chloropyrazin-2-yl)-3-methoxybenzamide (**2f**). White solid. Yield: 79%. mp 124.2–125.7 °C. ^{1}H NMR (500 MHz, DMSO-d_6) δ 11.31 (s, 1H, NH), 9.26 (d, *J* = 1.4 Hz, 1H, H3′), 8.63 (d, *J* = 1.4 Hz, 1H, H6′), 7.66–7.56 (m, 2H, ArH), 7.44 (t, *J* = 7.9 Hz, 1H, ArH), 7.18 (dd, *J* = 8.2, 2.6 Hz, 1H, ArH), 3.84 (s, 3H, OCH_3). ^{13}C NMR (126 MHz, DMSO-d_6) δ 166.03, 159.31, 148.22, 142.24, 141.98, 136.48, 134.61, 129.76, 120.70, 118.80, 113.12, 55.55. IR (ATR-Ge, cm^{-1}): 3379; 3125; 3100; 2939; 2836; 1680 (C=O, amide); 1585; 1530; 1513; 1484; 1429 1348; 1287; 1272; 1257; 1143; 1048; 1017; 809; 735; 677. Anal. calcd. for $C_{12}H_{10}ClN_3O_2$ (MW 263.68): C, 54.66; H, 3.82; N, 15.94. Found: C, 54.65; H, 3.825; N, 15.735.

N-(5-chloropyrazin-2-yl)-4-methoxybenzamide (**2g**). White solid. Yield: 83%. mp 177.8–179.0 °C. ^{1}H NMR (500 MHz, DMSO-d_6) δ 11.14 (s, 1H, NH), 9.24 (d, *J* = 1.5 Hz, 1H, H3′), 8.60 (s, 1H, H6′), 8.06 (d, *J* = 8.8 Hz, 2H, H2, H6), 7.05 (d, *J* = 8.8 Hz, 2H, H3, H5), 3.84 (s, 3H ,OCH_3). ^{13}C NMR (126 MHz, DMSO-d_6) δ 165.60, 162.74, 148.44, 142.12, 141.66, 136.41, 130.50, 125.25, 113.87, 55.66. IR (ATR-Ge, cm^{-1}): 3384; 3020; 2937; 2841; 1664 (C=O, amide); 1607; 1578; 1543 1495; 1441 1263; 1239; 1188; 1137; 1093; 1026; 1015; 867; 852; 760 . Anal. calcd. for $C_{12}H_{10}ClN_3O_2$ (MW 263.68): C, 54.66; H, 3.82; N, 15.94. Found: C, 54.655; H, 3.855; N, 15.695.

N-(5-chloropyrazin-2-yl)-4-methylbenzamide (**2h**). White solid. Yield: 77%. mp 186.4–187.1 °C. ^{1}H NMR (300 MHz, DMSO-d_6) δ 11.23 (s, 1H, NH), 9.25 (d, *J* = 1.5 Hz, 1H, H3′), 8.61 (d, *J* = 1.4 Hz, 1H, H6′), 7.96 (d, *J* = 8.1 Hz, 2H, H2, H6), 7.33 (d, *J* = 7.9 Hz, 2H, H3, H5), 2.38 (s, 3H, CH_3). ^{13}C NMR (75 MHz, DMSO-d_6) δ 166.17, 148.35, 142.83, 142.23, 141.84, 136.44, 130.45, 129.18, 128.48, 21.26. IR (ATR-Ge, cm^{-1}): 3336; 2921; 2856; 1654 (C=O, amide); 1541; 1497; 1442; 1340; 1283; 1251; 1137; 1100; 1065; 1019; 869; 837; 748; 709; 692. Anal. calcd. for $C_{12}H_{10}ClN_3O$ (MW 247.68): C, 58.19; H, 4.07; N, 16.97. Found: C, 58.23; H, 4.13; N, 16.675.

N-(5-chloropyrazin-2-yl)-4-ethylbenzamide (**2i**). White solid. Yield: 85%. mp 150.0–150.8 °C. ^{1}H NMR (500 MHz, DMSO-d_6) δ 11.22 (s, 1H, NH), 9.26 (d, *J* = 1.5 Hz, 1H, H3′), 8.61 (d, *J* = 1.4 Hz, 1H, H6′), 7.98 (d, *J* = 8.3 Hz, 2H, H2, H6), 7.36 (d, *J* = 8.1 Hz, 2H, H3, H5), 2.68 (q, *J* = 7.6 Hz, 2H, CH_2), 1.21 (t, *J* = 7.6 Hz, 3H, CH_3). ^{13}C NMR (126 MHz, DMSO-d_6) δ 166.18, 148.88, 148.32, 142.20, 141.82, 136.41, 130.72, 128.55, 127.99, 28.27, 15.39. IR (ATR-Ge, cm^{-1}): 3356; 2972; 2928; 1670 (C=O, amide); 1539; 1493; 1339; 1247; 1132; 1104; 1015; 905; 869; 846. Anal. calcd. for $C_{13}H_{12}ClN_3O$ (MW 261.71): C, 59.66; H, 4.62; N, 16.06. Found: C, 59.46; H, 4.61; N, 15.8.

2-chloro-*N*-(5-chloropyrazin-2-yl)benzamide (**2j**). White solid. Yield: 68%. mp 158.9–159.7 °C. ^{1}H NMR (500 MHz, DMSO-d_6) δ 11.58 (s, 1H,NH), 9.25 (d, *J* = 1.5 Hz, 1H, H3′), 8.60 (d, *J* = 1.4 Hz, 1H, H6′), 7.62 (dd, *J* = 7.5, 1.7 Hz, 1H , ArH), 7.59–7.50 (m, 2H ,ArH), 7.46 (td, *J* = 7.4, 1.4 Hz, 1H, ArH). ^{13}C NMR (126 MHz, DMSO-d_6) δ 166.01, 147.69, 142.56, 142.29, 135.69, 131.78, 130.15, 129.83, 129.34, 127.34. IR (ATR-Ge, cm^{-1}): 3208; 3064; 1687 (C=O, amide); 1541; 1496; 1433; 1335; 1293; 1258; 1122; 1020; 774; 745; 684; 657. Anal. calcd. for $C_{11}H_7Cl_2N_3O$ (MW 268.1): C, 49.28; H, 2.63; N, 15.67. Found: C, 49.53; H, 2.63; N, 15.47.

3-chloro-*N*-(5-chloropyrazin-2-yl)benzamide (**2k**). White solid. Yield: 54%. mp 150.7–151.9 °C. ^{1}H NMR (500 MHz, DMSO-d_6) δ 11.44 (s, 1H, NH), 9.23 (d, *J* = 1.4 Hz, 1H, H3′), 8.63 (d, *J* = 1.4 Hz, 1H, H6′), 8.08 (t, *J* = 1.9 Hz, 1H, H2), 8.01–7.95 (m, 1H, H6), 7.71–7.66 (m, 1H, H4), 7.56 (t, *J* = 7.9 Hz, 1H, H5). ^{13}C NMR (126 MHz, DMSO-d_6) δ 165.00, 148.02, 142.32, 142.18, 136.43, 135.31, 133.41,

132.32, 130.55, 128.21, 127.13. IR (ATR-Ge, cm^{-1}): 3366; 3074; 2925; 1672 (C=O, amide); 1541; 1506; 1473; 1345; 1289; 1243; 1136; 1018; 763; 741; 692; 677. Anal. calcd. for $C_{11}H_7Cl_2N_3O$ (MW 268.1): C, 49.28; H, 2.63; N, 15.67. Found: C, 49.29; H, 2.71; N, 15.375.

4-chloro-*N*-(5-chloropyrazin-2-yl)benzamide (**2l**). White solid. Yield: 8%. mp 225.0–226.3 °C. ^{1}H NMR (500 MHz, DMSO-d_6) δ 11.41 (s, 1H, NH), 9.24 (d, *J* = 1.5 Hz, 1H, H3′), 8.63 (d, *J* = 1.6 Hz, 1H, H6′), 8.05 (d, *J* = 8.5 Hz, 2H, H2, H6), 7.60 (d, *J* = 8.5 Hz, 2H, H3, H5). ^{13}C NMR (126 MHz, DMSO-d_6) δ 165.37, 148.11, 142.29, 142.09, 137.47, 136.44, 132.09, 130.36, 128.69. IR (ATR-Ge, cm^{-1}): 3374; 3127; 3101; 1679 (C=O, amide); 1530; 1515; 1480; 1440; 1344; 1295; 1249; 1136; 1097; 1016; 840; 746. Anal. calcd. for $C_{11}H_7Cl_2N_3O$ (MW 268.1): C, 49.28; H, 2.63; N, 15.67. Found: C, 49.44; H, 2.565; N, 15.585.

4-bromo-*N*-(5-chloropyrazin-2-yl)benzamide (**2m**). White solid. Yield: 43%. mp 229.2–230.5 °C. ^{1}H NMR (500 MHz, DMSO-d_6) δ 11.42 (s, 1H, NH), 9.24 (d, *J* = 1.4 Hz, 1H, H3′), 8.63 (d, *J* = 1.4 Hz, 1H, H6′), 7.97 (d, *J* = 8.6 Hz, 2H, H2, H6), 7.74 (d, *J* = 8.5 Hz, 2H, H3, H5). ^{13}C NMR (126 MHz, DMSO-d_6) δ 165.52, 148.11, 142.31, 142.10, 136.45, 132.47, 131.65, 130.51, 126.51. IR (ATR-Ge, cm^{-1}): 3313; 1650 (C=O, amide); 1544; 1514; 1484; 1335; 1287; 1139; 1105; 1072; 1021; 1007; 868; 848; 658. Anal. calcd. for $C_{11}H_7BrClN_3O$ (MW 312.55): C, 42.27; H, 2.26; N, 13.44. Found: C, 42.23; H, 2.27; N, 13.29.

N-(5-chloropyrazin-2-yl)-3-(trifluoromethyl)benzamide (**2n**). White solid. Yield: 65%. mp 155.4–156.4 °C. ^{1}H NMR (500 MHz, DMSO-d_6) δ 11.62 (s, 1H, NH), 9.26 (d, *J* = 1.4 Hz, 1H. H3′), 8.64 (d, *J* = 1.3 Hz, 1H, H6′), 8.40 (bs, 1H, H2), 8.31 (d, *J* = 8.2 Hz, 1H H6), 7.98 (d, *J* = 7.8 Hz, 1H, H4), 7.78 (t, *J* = 7.8 Hz, 1H, H5). ^{13}C NMR (126 MHz, DMSO-d_6) δ 165.00, 148.04, 142.34, 142.24, 136.45, 134.27, 132.52, 129.90, 129.35 (q, *J* = 32.1 Hz), 129.02 (q, *J* = 3.8 Hz), 125.13 (q, *J* = 3.9 Hz), 124.05 (q, *J* = 272.6 Hz). IR (ATR-Ge, cm^{-1}): 3367; 1679 (C=O, amide); 1541; 1512; 1454; 1329;1237; 1181; 1131; 1073; 1018; 916; 791; 750; 720; 694; 669. Anal. calcd. for $C_{12}H_7ClF_3N_3O$ (MW 301.65): C, 47.78; H, 2.34; N, 13.93. Found: C, 48.23; H, 2.25; N, 13.78.

N-(6-chloropyrazin-2-yl)benzamide (**3a**). White to pale beige solid. Yield: 28%. mp 152.8-153.6 °C. ^{1}H NMR (500 MHz, DMSO-d_6) δ 11.41 (bs, 1H, NH), 9.42 (s, 1H, H3′), 8.53 (s, 1H, H5′), 8.07–8.03 (m, 2H, H2, H6), 7.66–7.60 (m, 1H, H4), 7.53 (m, 2H , H3, H5). ^{13}C NMR (126 MHz, DMSO-d_6) δ 166.39, 148.65, 145.58, 138.64, 135.30, 133.12, 132.70, 128.61, 128.48. IR (ATR-Ge, cm^{-1}): 3209; 3066; 1686 (C=O, amide); 1542; 1398; 1292; 1267; 1164; 1008; 882. Anal. calcd. for $C_{11}H_8ClN_3O$ (MW 233.66): C, 56.55; H, 3.45; N, 17.98. Found: C, 56.59; H, 3.42; N, 17.85.

N-(6-chloropyrazin-2-yl)-2-methoxybenzamide (**3e**). White to pale beige solid. Yield: 20%. mp 149.1-150.7 °C. ^{1}H NMR (500 MHz, DMSO-d_6) δ 10.86 (bs, 1H, NH), 9.44 (s, 1H, H3′), 8.53 (s, 1H, H5′), 7.78 (dd, *J* = 7.7, 1.7 Hz, 1H, H6), 7.62–7.54 (m, 1H, H4), 7.23 (d, *J* = 8.4 Hz, 1H, H3), 7.10 (t, *J* = 7.5 Hz, 1H, H5), 3.95 (s, 3H, OCH_3). ^{13}C NMR (126 MHz, DMSO-d_6) δ 164.88, 157.23, 147.92, 145.69, 138.82, 134.50, 133.69, 130.57, 122.32, 120.91, 112.50, 56.39. IR (ATR-Ge, cm^{-1}): 3320; 3071; 2951; 1676 (C=O, amide); 1526; 1483; 1395; 1282; 1152; 1012; 1001; 756; 673. Anal. calcd. for $C_{12}H_{10}ClN_3O_2$ (MW 263.68): C, 54.66; H, 3.82; N, 15.94. Found: C, 54.28; H, 3.64; N, 15.61.

N-(6-chloropyrazin-2-yl)-3-methoxybenzamide (**3f**). White to pale beige solid. Yield: 27%. mp 131.6-132.7 °C. ^{1}H NMR (500 MHz, DMSO-d_6) δ 11.41 (s, 1H, NH), 9.42 (s, 1H, H3′), 8.53 (s, 1H, H5′), 7.67–7.58 (m, 2H, ArH), 7.44 (t, *J* = 7.9 Hz, 1H, ArH), 7.18 (dd, *J* = 8.2, 2.5 Hz, 1H, ArH), 3.84 (s, 3H, OCH_3). ^{13}C NMR (126 MHz, DMSO-d_6) δ 166.07, 159.31, 148.62, 145.57, 138.67, 135.33,

134.41, 129.76, 120.80, 119.03, 113.09, 55.56. IR (ATR-Ge, cm^{-1}): 3228; 3072; 2947; 2836; 1686 (C=O, amide); 1541; 1488; 1415; 1398; 1283; 1160; 1047; 1007; 733; 704; 680. Anal. calcd. for $C_{12}H_{10}ClN_3O_2$ (MW 263.68): C, 54.66; H, 3.82; N, 15.94. Found: C, 54.36; H, 3.72; N, 15.7.

N-(6-chloropyrazin-2-yl)-4-methoxybenzamide (**3g**). White to pale beige solid. Yield: 8%. mp 196.9-198.8 °C. ^{1}H NMR (500 MHz, DMSO-*d*$_6$) δ 11.25 (bs, 1H, NH), 9.41 (s, 1H, H3′), 8.51 (s, 1H, H5′), 8.07 (d, *J* = 8.8 Hz, 2H, H2, H6), 7.06 (d, *J* = 8.9 Hz, 2H, H3, H5), 3.85 (s, 3H, OCH_3). ^{13}C NMR (126 MHz, DMSO-*d*$_6$) δ 165.64, 162.85, 148.86, 145.52, 138.30, 135.29, 130.60, 125.08, 113.90, 55.69. IR (ATR-Ge, cm^{-1}): 3065; 2922; 2843; 1679 (C=O, amide); 1550; 1509; 1416; 1399; 1260; 1187; 1164; 1008; 838; 738; 694. Anal. calcd. for $C_{12}H_{10}ClN_3O_2$ (MW 263.68): C, 54.66; H, 3.82; N, 15.94. Found: C, 54.83; H, 3.6; N, 15.79.

N-(6-chloropyrazin-2-yl)-4-methylbenzamide (**3h**). White to pale beige solid. Yield: 29%. mp 169.1-170.0 °C. ^{1}H NMR (500 MHz, DMSO-*d*$_6$) δ 11.32 (bs, 1H, NH), 9.41 (s, 1H, H3′), 8.51 (s, 1H, H5′), 7.97 (d, *J* = 8.2 Hz, 2H, H2, H6), 7.33 (d, *J* = 7.9 Hz, 2H, H3, H5), 2.38 (s, 3H, CH_3). ^{13}C NMR (126 MHz, DMSO-*d*$_6$) δ 166.19, 148.73, 145.55, 142.95, 138.48, 135.29, 130.27, 129.16, 128.53, 21.24. IR (ATR-Ge, cm^{-1}): 3205; 3063; 1684 (C=O, amide); 1541; 1398; 1293; 1271; 1159; 1106; 1008; 831; 736; 689. Anal. calcd. for $C_{12}H_{10}ClN_3O$ (MW 247.68): C, 58.19; H, 4.07; N, 16.97. Found: C, 57.89; H, 3.99; N, 16.53.

N-(6-chloropyrazin-2-yl)-4-ethylbenzamide (**3i**). White to pale beige solid. Yield: 26%. mp 142.5-143.3 °C. ^{1}H NMR (500 MHz, DMSO-*d*$_6$) δ 11.33 (bs, 1H, NH), 9.42 (s, 1H, H3′), 8.52 (s, 1H, H5′), 7.99 (d, *J* = 8.3 Hz, 2H, H2, H6), 7.37 (d, *J* = 8.3 Hz, 2H, H3, H5), 2.69 (q, *J* = 7.6 Hz, 2H, CH_2), 1.21 (t, *J* = 7.6 Hz, 3H, CH_3). ^{13}C NMR (126 MHz, DMSO-*d*$_6$) δ 166.21, 149.04, 148.73, 145.55, 138.50, 135.29, 130.53, 128.63, 128.00, 28.27, 15.36. IR (ATR-Ge, cm^{-1}): 3203; 3063; 2969; 2882; 1682 (C=O, amide); 1545; 1398; 1293; 1271; 1152; 1008; 845; 726. Anal. calcd. for $C_{13}H_{12}ClN_3O$ (MW 261.71): C, 59.66; H, 4.62; N, 16.06. Found: C, 59.22; H, 4.49; N, 15.74.

2-chloro-*N*-(6-chloropyrazin-2-yl)benzamide (**3j**). White to pale beige solid. Yield: 23%. mp 182.4-183.4 °C. ^{1}H NMR (500 MHz, DMSO-*d*$_6$) δ 11.67 (s, 1H, NH), 9.40 (s, 1H, H3′), 8.56 (s, 1H, H5′), 7.62 (dd, *J* = 7.5, 1.8 Hz, 1H, ArH), 7.59–7.50 (m, 2H, ArH), 7.49–7.42 (m, 1H, ArH). ^{13}C NMR (126 MHz, DMSO-*d*$_6$) δ 166.07, 147.98, 145.76, 139.14, 135.46, 134.59, 131.88, 130.17, 129.84, 129.37, 127.32. IR (ATR-Ge, cm^{-1}): 3145; 3008; 2928; 1699 (C=O, amide); 1537; 1417; 1401; 1287; 1152; 1124; 1005; 873; 745. Anal. calcd. for $C_{11}H_7Cl_2N_3O$ (MW 268.1): C, 49.28; H, 2.63; N, 15.67. Found: C, 49.1; H, 2.49; N, 15.37.

3-chloro-*N*-(6-chloropyrazin-2-yl)benzamide (**3k**). White to pale beige solid. Yield: 32%. mp 166.0-167.1 °C. ^{1}H NMR (500 MHz, DMSO-*d*$_6$) δ 11.53 (s, 1H, NH), 9.40 (s, 1H, H3′), 8.55 (s, 1H, H5′), 8.10 (t, *J* = 1.9 Hz, 1H, H2), 8.02–7.96 (m, 1H, H6), 7.71–7.68 (m, 1H, ArH), 7.56 (t, *J* = 7.9 Hz, 1H, ArH). ^{13}C NMR (126 MHz, DMSO-*d*$_6$) δ 165.05, 148.40, 145.61, 138.91, 135.26, 135.13, 133.43, 132.45, 130.57, 128.29, 127.20. IR (ATR-Ge, cm^{-1}): 3202; 3070; 1691 (C=O, amide); 1542; 1415; 1397; 1296; 1256; 1166; 1009; 775; 732; 690. Anal. calcd. for $C_{11}H_7Cl_2N_3O$ (MW 268.1): C, 49.28; H, 2.63; N, 15.67. Found: C, 49.45; H, 2.47; N, 15.63.

4-chloro-*N*-(6-chloropyrazin-2-yl)benzamide (**3l**). White to pale beige solid. Yield: 36%. mp 193.8-194.7 °C. ^{1}H NMR (500 MHz, DMSO-*d*$_6$) δ 11.50 (s, 1H, NH), 9.40 (s, 1H, H3′), 8.54 (s, 1H, H5′), 8.06 (d, *J* = 8.6 Hz, 2H, H2, H6), 7.60 (d, *J* = 8.7 Hz, 2H, H3, H5). ^{13}C NMR (126 MHz, DMSO-*d*$_6$) δ

165.40, 148.50, 145.58, 138.79, 137.60, 135.27, 131.92, 130.43, 128.70. IR (ATR-Ge, cm^{-1}): 3196; 3065; 1687 (C=O, amide); 1546; 1401; 1292; 1268; 1167; 1103; 1009; 842; 750; 682. Anal. calcd. for $C_{11}H_7Cl_2N_3O$ (MW 268.1): C, 49.28; H, 2.63; N, 15.67. Found: C, 49.41; H, 2.54; N, 15.56.

N-(6-chloropyrazin-2-yl)-3-(trifluoromethyl)benzamide (**3n**). White to pale beige solid. Yield: 46%. mp 124.2—125.0 °C. ^{1}H NMR (500 MHz, DMSO-*d*$_6$) δ 11.70 (bs, 1H, NH), 9.42 (s, 1H, H3′), 8.55 (s, 1H, H5′), 8.42 (bs, 1H, H2), 8.31 (d, *J* = 7.8 Hz, 1H, H6), 7.99 (d, *J* = 7.8 Hz, 1H, H4), 7.77 (t, *J* = 7.8 Hz, 1H, H5). ^{13}C NMR (126 MHz, DMSO-*d*$_6$) δ 165.05, 148.42, 145.61, 138.96, 135.25, 134.08, 132.59, 129.92, 129.35 (q, *J* = 32.3 Hz), 129.14 (q, *J* = 3.8 Hz), 125.22 (q, *J* = 3.8 Hz), 124.04 (q, *J* = 272.6 Hz). IR (ATR-Ge, cm^{-1}): 3303; 3040; 1655 (C=O, amide); 1537; 1400; 1330; 1256; 1157; 1118; 1076; 1008; 826; 702; 677. Anal. calcd. for $C_{12}H_7ClF_3N_3O$ (MW 301.65): C, 47.78; H, 2.34; N, 13.93. Found: C, 47.55; H, 2.01; N, 13.87.

2.2. Evaluation of *in vitro* antimycobacterial activity

A microdilution panel method was used. Tested strains *Mycobacterium tuberculosis* H37Rv CNCTC My 331/88 (ATCC 27294), *M. kansasii* CNCTC My 235/80 (ATCC12478) and *M. avium* ssp. *avium* CNCTC My 80/72 (ATCC 15769) were obtained from the Czech National Collection of Type Cultures (CNCTC), National Institute of Public Health (Prague, Czech Republic). Middlebrook 7H9 broth (Sigma-Aldrich, Steinheim, Germany) enriched with the 0.4% of glycerol (Sigma-Aldrich) and 10% of OADC supplement (oleic acid, albumin, dextrose, catalase; Himedia, Mumbai, India) of declared pH = 6.6 was used for cultivation. Tested compounds were dissolved and diluted in DMSO and mixed with broth (25 μL of DMSO solution in 4.475 mL of broth) and placed (100 μL) into microplate wells. Mycobacterial inocula were suspended in isotonic saline solution and the density was adjusted to 0.5–1.0 McFarland. These suspensions were diluted by 10^{-1} and used to inoculate the testing wells, adding 100 μL of suspension to 100 μL of the DMSO/broth solution of tested compound. Final concentrations of tested compounds in wells were 100, 50, 25, 12.5, 6.25, 3.13 and 1.56 $\mu g \cdot mL^{-1}$. Isoniazid (INH) was used as positive control (inhibition of growth). Negative control consisted of broth plus DMSO. A total of 30 μL of Alamar Blue working solution (1:1 mixture of 0.01% resazurin sodium salt (aq. sol.) and 10% Tween 80) was added after five days of incubation. Results were then determined after 24 h of incubation. The minimum inhibitory concentration (MIC; $\mu g \cdot mL^{-1}$) was determined as the lowest concentration that prevented the blue to pink colour change. MIC values of INH were 6.25–12.5 $\mu g \cdot mL^{-1}$ against *M. avium*, 3.13–12.5 $\mu g \cdot mL^{-1}$ against *M. kansasii*, and 0.1–0.2 $\mu g \cdot mL^{-1}$ against *M. tbc*.

2.3. Antimycobacterial i*n vitro* activity screening against *Mycobacterium smegmatis*

The antimycobacterial assay was performed with fast growing *Mycobacterium smegmatis* CCM 4622 (ATCC 607) from the Czech Collection of Microorganisms (Brno, Czech Republic). The technique used for activity determination was microdilution broth panel method using 96-well microtitration plates. The culturing medium was Middlebrook 7H9 (MB) broth (Sigma-Aldrich), enriched with 0.4% of glycerol (Sigma-Aldrich, Steinheim, Germany) and 10% of Middlebrook OADC growth supplement (Himedia). Tested compounds were dissolved in DMSO (Sigma-Aldrich), and the MB broth was then added to achieve a concentration of 2000 $\mu g \cdot mL^{-1}$. Standards

used for activity determination were INH, rifampicin (RIF), and ciprofloxacin (CPX) (Sigma-Aldrich). Final concentrations were reached by binary dilution followed by the addition of mycobacterial suspension, and were set as 500, 250, 125, 62.5, 31.25, 15.625, 7.81, and 3.91 μg·mL^{-1}, except for the standards of ciprofloxacin and rifampicin, where the final concentrations were 12.5, 6.25, 3.125, 1.56, 0.78, 0.39, 0.195, and 0.098 μg·mL^{-1}. The final concentration of DMSO did not exceeded 2.5% (*v*/*v*) and did not affect the growth of *M. smegmatis*. Plates were also sealed with polyester adhesive film and incubated in the dark at 37 °C, without agitation. The addition of 0.01% solution of resazurin sodium salt followed after 48 h. This stain was prepared by dissolving resazurin sodium salt (Sigma-Aldrich) in deionised water, producing a 0.02% solution. Then, a 10% aqueous solution of Tween 80 (Sigma-Aldrich) was prepared. Both liquids were mixed up making use of the same volumes and filtered through a syringe membrane filter. Microtitration panels were then further incubated for 4 h. Antimycobacterial activity was expressed as the minimal inhibition concentration (MIC) and the value was read on the basis of stain colour change (blue colour—active compound; pink colour—not active compound). The MIC values for the standards were in the range of 7.81–15.625 μg·mL^{-1} for INH, 0.78–1.56 μg·mL^{-1} for RIF, and 0.098–0.195 μg·mL^{-1} for CPX. All experiments were conducted in duplicate.

2.4. Evaluation of i*n vitro* antibacterial activity

Microdilution broth method was used. Antibacterial evaluation was performed against eight bacterial strains from the Czech Collection of Microorganisms (*Staphylococcus aureus* CCM 4516/08, *Escherichia coli* CCM 4517, *Pseudomonas aeruginosa* CCM 1961) or clinical isolates from the Department of Clinical Microbiology, University Hospital and Faculty of Medicine in Hradec Králové, Charles University in Prague, Czech Republic (*Staphylococcus aureus* H 5996/08-methicilin resistant (MRSA), *Staphylococcus epidermidis* H 6966/08, *Enterococcus* sp. J 14365/08, *Klebsiella pneumoniae* D 11750/08, *Klebsiella pneumoniae* J 14368/08-ESBL positive). All strains were subcultured on Mueller-Hinton agar (MHA) (Difco/Becton Dickinson, Detroit, MI, USA) at 35 °C and maintained on the same medium at 4 °C. The compounds were dissolved in DMSO, and the antibacterial activity was determined in Mueller-Hinton liquid broth (Difco/Becton Dickinson), and buffered to pH 7.0. Controls consisted of medium and DMSO alone. The final concentration of DMSO in the test medium did not exceed 1% (*v*/*v*) of the total solution composition. The minimum inhibitory concentration (MIC), defined as the minimum concentration to prevent the visible growth compared to control, was determined after 24 and 48 h of static incubation at 35 °C. The standards were neomycin, bacitracin, penicillin G, ciprofloxacin, and phenoxymethylpenicillin.

2.5. Evaluation of i*n vitro* antifungal activity

Antifungal evaluation was performed using a microdilution broth method against eight fungal strains (*Candida albicans* ATCC 44859, *C. tropicalis* 156, *C. krusei* E28, *C. glabrata* 20/I, *Trichosporon asahii* 1188, *Aspergillus fumigatus* 231, *Lichtheimia corymbifera* 272 and *Trichophyton interdigitale* 445). Compounds were dissolved in DMSO and diluted in a twofold manner with RPMI 1640 medium, with glutamine buffered to pH 7.0 (3-morpholinopropane-1-sulfonic acid). The final concentration of DMSO in the tested medium did not exceed 2.5% (v/v) of the total solution composition. Static incubation was performed in the dark and humidity, at 35 °C, for 24 and 48 h

(72 and 120 h for *Trichophyton interdigitale*). Drug-free controls were included. The standards were amphotericin B, voriconazole, nystatin, and fluconazole.

2.6. HepG2 cytotoxicity determination

The human liver hepatocellular carcinoma cell line HepG2 (passage 32–34 for compounds **2b-Ac**, **2b**, **2h**, and **2i**; and passage 17–18 for **2d-Ac** and **3n**) purchased from Health Protection Agency Culture Collections (ECACC, Salisbury, UK) was routinely cultured in Minimum Essential Eagle Medium (MEM; Sigma-Aldrich) supplemented with 10% (v/v) fetal bovine serum (PAA, Austria), 1% (v/v) L-glutamine solution (Sigma-Aldrich) and 1% (v/v) non-essential amino acid solution (Sigma-Aldrich) in a humidified atmosphere containing 5% CO_2 at 37 °C. For subculturing, the cells were harvested after trypsin/EDTA (Sigma-Aldrich) treatment at 37°C. To evaluate the cytotoxicity, the HepG2 cells treated with the tested substances were used as experimental groups whereas untreated HepG2 cells served as control groups.

HepG2 cells were seeded in a density of $1×10^4$ cells per well on a 96-well plate. Next day (24 h after seeding) the cells were treated with tested substances dissolved in DMSO at different concentrations ranging from 0.1 to 1000 μM (depending on the solubility, see Table 2). Maximal incubation concentration of DMSO in a well did not exceed 1% (v/v). The treatment was carried out in triplicates in a humidified atmosphere containing 5% of CO_2 at 37 °C. The controls representing 100% cell viability (untreated cells), 0% cell viability (cells treated with 10% DMSO), no-cell controls and vehiculum controls were incubated in triplicates simultaneously. After 24 h exposure to tested compounds, CellTiter 96® Aqueous One Solution Cell Proliferation Assay (Promega, Madison, WI, USA) reagent was added to each well according to the manufacturer`s recommendations. After 2 h incubation at 37 °C in humidified, 5% of CO_2 containing atmosphere, the absorbance was recorded at 490 nm. Inhibitory curves were constructed for each compound plotting incubation concentrations *vs.* percentage of absorbance relative to untreated control. The standard toxicological parameter IC_{50} was calculated by nonlinear regression analysis of the inhibitory curves using GraphPad Prism software, version 6 (GraphPad Software, Inc., CA, USA).

2.7. Confirmatory test of HepG2 cytotoxicity

The HepG2 cells (passage 35–36) were seeded in density $1×10^4$ cells per well on a 96-well plate. Next day (24 h after seeding), they were treated with tested substances dissolved in DMSO (maximal incubation concentration of DMSO was 1% v/v). The tested substances were prepared according to their solubility in DMSO at incubation concentrations 0.1–500 μM (see Table 3). The treatment was carried out in a humidified atmosphere containing 5% CO_2 at 37 °C in triplicates for 24 h and 48 h. The controls representing 100% cell viability, 0% cell viability (the cells treated with 10% DMSO and the cells treated with Lysis Solution 1:25), no-cell controls and vehiculum controls were incubated in triplicates simultaneously. After 24 h exposure, the reagent from the kit CellTox™ Green Cytotoxicity Assay (Promega, Madison, WI, USA) was prepared and added according to the recommendation of the manufacturer. After 15 min incubation at room

temperature, the fluorescence was measured at $485nm_{Ex}/520nm_{Em}$. The measurement was repeated after 24 h to get results for the exposure period 48 h. Inhibitory curves were constructed for each compound plotting incubation concentrations vs. percentage of fluorescence relative to untreated control. The standard toxicological parameter IC_{50} was calculated by nonlinear regression analysis of the inhibitory curves using GraphPad Prism software version 6 (GraphPad Software, Inc., CA, USA).

References:

1. Matyk, J.; Waisser, K.; Drazkova, K.; Kunes, J.; Klimesova, V.; Palat, K., Jr.; Kaustova, J. Heterocyclic isosters of antimycobacterial salicylanilides. *Farmaco* **2005,** *60*, 399-408.
2. Kakemi, K.; Arta, T.; Kitazawa, S.; Kiyotaki, T. [Studies on the synthesis of pyrazinoic acid derivatives. II. Derivatives of 3-aminopyrazinoic acid]. *Yakugaku Zasshi* **1961,** *81*, 1650-3.
3. Hachiya, S.; Inagaki, T.; Hashizume, D.; Maki, S.; Niwa, H.; Hirano, T. Synthesis and fluorescence properties of difluoro[amidopyrazinato-O,N]boron derivatives: a new boron-containing fluorophore. *Tetrahedron Lett.* **2010,** *51*, 1613 - 1615.
4. Sambaiah, T.; Reddy, K. K. Synthesis of 2-aryl-<1,2,4>triazolo-<1,5-a>pyrazines. *Indian Journal of Chemistry, Section B: Organic Chemistry Including Medicinal Chemistry* **1992,** *31*, 444 - 445.
5. Vigorita; Grasso; Zappala; Ottana; Monforte; Barbera; Trovato. Aminopyrazinyl derivatives: Synthesis and evaluation of antiinflammatory and related activities. *Farmaco* **1994,** *49*, 271 - 276.
